# Symmetrical treatment of “*Diagnostic and Statistical Manual of Mental Disorders*, Fifth Edition”, for major depressive disorders

**DOI:** 10.1186/s13029-015-0041-7

**Published:** 2016-01-19

**Authors:** Jitsuki Sawamura, Shigeru Morishita, Jun Ishigooka

**Affiliations:** Department of Psychiatry, Tokyo Women’s Medical University, Tokyo, Japan; Depression Prevention Medical Center, Inariyama Takeda Hospital, Kyoto, Japan

**Keywords:** DSM-5, Diagnosis, Criterion, Depression, Abstract algebra, Upgrading

## Abstract

**Background:**

We previously presented a group theoretical model that describes psychiatric patient states or clinical data in a graded vector-like format based on modulo groups. Meanwhile, the *Diagnostic and Statistical Manual of Mental Disorders*, Fifth Edition (DSM-5, the current version), is frequently used for diagnosis in daily psychiatric treatments and biological research. The diagnostic criteria of DSM-5 contain simple binominal items relating to the presence or absence of specific symptoms. In spite of its simple form, the practical structure of the DSM-5 system is not sufficiently systemized for data to be treated in a more rationally sophisticated way. To view the disease states in terms of symmetry in the manner of abstract algebra is considered important for the future systematization of clinical medicine.

**Results:**

We provide a simple idea for the practical treatment of the psychiatric diagnosis/score of DSM-5 using depressive symptoms in line with our previously proposed method. An expression is given employing modulo-2 and −7 arithmetic (in particular, additive group theory) for Criterion A of a ‘major depressive episode’ that must be met for the diagnosis of ‘major depressive disorder’ in DSM-5. For this purpose, the novel concept of an imaginary value 0 that can be recognized as an explicit 0 or implicit 0 was introduced to compose the model. The zeros allow the incorporation or deletion of an item between any other symptoms if they are ordered appropriately. Optionally, a vector-like expression can be used to rate/select only specific items when modifying the criterion/scale. Simple examples are illustrated concretely.

**Conclusions:**

Further development of the proposed method for the criteria/scale of a disease is expected to raise the level of formalism of clinical medicine to that of other fields of natural science.

## Background

Group theory is one of the cornerstones of various branches of natural science [1–8]. Unfortunately, clinical medicine including psychiatry has not been optimally systematized, or attained a level of formalism, sophisticated enough to be linked directly with other fields of natural science. Additionally, it is considered important to view disease states in terms of symmetry in the manner of abstract algebra. We previously addressed this issue by presenting patient states or clinical data in a graded vector-like format based on modulo groups [9, 10]. In that work, we briefly demonstrated modulo-p (p: prime) arithmetic, particularly in the case p = 7. The operator A_(j→k)_ acting on the state A_j_ follows the right-translation rule denoted † (with * denoting collective addition); i.e., the operator acts from the right side of the state as in A_j_ † A_(j→k)_ = A_k_. The disease states and clinical data were entirely treated using operations. As a practical application, we presume that this method can be simply applied to, for example, an operational diagnosing system such as that in *Diagnostic and Statistical Manual of Mental Disorders* (DSM) (where the fifth edition, DSM-5, is the current version) [11].

DSM-5 is widely used around the world, mainly by psychiatrists in biological fields for basic biological (e.g., pharmacological, molecular biological, and genetic) and cognitive-behavioral/psychoeducational therapies. The first edition of the DSM (DSM-I) was published in 1952 by the American Psychiatric Association and has since been revised; DSM-II was published in 1968, DMS-III in 1980, DSM-III-R (Revision) in 1987, DSM-IV in 1994, DSM-IV-TR (Text Revision) in 2000 [12] and DSM-5 in 2013 [11]. In the revisions, standardized criteria were added and the number of adopted diseases increased enormously. In particular, from DSM-III onwards, the diagnostic criteria in the DSM contained more details of each symptom in binominal form (e.g., presence/absence) for the diagnosis of mental disorders by psychiatrists. For instance, Criterion A for a ‘major depressive episode’ that needs to be met for the diagnosis of a ‘major depressive disorder’ in DSM-5 comprises nine specific symptoms: (1) ‘depressed mood most of the day’; (2) ‘markedly diminished interest or pleasure in all, or almost all, activities most of the day, nearly every day’; (3) ‘significant weight loss when not dieting or weight gain’; (4) ‘insomnia or hypersomnia nearly every day’; (5) ‘psychomotor agitation or retardation nearly every day’; (6) ‘fatigue or loss of energy every day’; (7) ‘feelings of worthlessness or excessive or inappropriate guilt’; (8) ‘diminished ability to think or concentrate, or indecisiveness, nearly every day’; and (9) ‘recurrent thoughts of death’. Criterion A requires five (or more) of the symptoms to have been present during the same 2-week period and to represent a change from previous functioning, and at least one of the symptoms must be either symptom (1) or symptom (2). Additionally, all of Criteria B, C, D and E need to be fulfilled to confirm a ‘major depressive episode’ [11]. This paper uses a fictional dataset to demonstrate a series of scenarios; 10 assessments (patients/sessions) that constitute the dataset are given in Table 1. In this manner, owing to the simple structure of DSM-III, almost all mental disorders are diagnosed operationally/manually. To our knowledge, this operational form of diagnosis has resulted in various confrontations among psychiatrists having various viewpoints (e.g., viewpoints relating to biology, psychopharmacology, psychopathology, and psychology). Until now, these conflicts have appeared to be irreconcilable.

**Table 1:** Presentation of examples of diagnostic assessment on Criterion A

Symptom number	1	2	3	4	5	6	7	8	9		Episode
row 1	1	1	1	0	1	1	0	1	1	(mod 2)	1
row 2	0	1	0	0	1	0	0	1	1	(mod 2)	0
row 3	0	0	0	1	0	0	0	1	0	(mod 2)	0
row 4	1	1	0	1	1	0	1	0	1	(mod 2)	1
row 5	0	1	1	1	0	0	0	0	1	(mod 2)	0
row 6	1	1	1	0	1	1	0	0	1	(mod 2)	1
row 7 (= row 4)	1	1	0	1	1	0	1	0	1	(mod 2)	1
row 8 (= row 2)	0	1	0	0	1	0	0	1	1	(mod 2)	1
row 9 (= row 5)	0	1	1	1	0	0	0	0	1	(mod 2)	1
row 10 (= row 4)	1	1	0	1	1	0	1	0	1	(mod 2)	1
Total sum	5	9	4	6	7	2	3	4	9		5
Average	0.5	0.9	0.4	0.6	0.7	0.2	0.3	0.4	0.9		0.5

Examples for symptoms 1–9 in Criterion A having values of 0 or 1 are shown. Each row is an assessment during a session. Rows 3 and 5 are equivalent to row 1; i.e., row = row 3 = row 5. Additionally, row 4 = row 6 = row 7 = row 9 = row 10, and row 2 = row 8. The expression of these examples can be simplified as in Table 2. In this case, the order of ‘which items should be effective on the scale’, is A_all(1–9)_ = [1_1_|1_2_|1_3_|1_4_|1_5_|1_6_|1_7_|1_8_|1_9_||**0**_**10**_|**0**_**11**_|**0**_**12**_|…] (mod 2); all symptoms 1–9 in Criterion A should be effective, and this could be reinterpreted as the result of the operation (selection for effectiveness) A_all(1–9)_ (= A_(0→all(1–9))_) acting on the identity order A_0_ = [0_1_|0_2_|0_3_|0_4_|0_5_|0_6_|0_7_|0_8_|0_9_||**0**_**10**_|**0**_**11**_|**0**_**12**_|…] (mod 2); i.e., A_0_ * A_(0→all(1–9))_ = A_all(1–9)_. A_0_ could be also regarded as an undiagnosed state. The rows whose components are equivalent to each other are compressed in the earliest rows of Table 2 and are highlighted silver in Table 1. Additionally, the diagnosis is given in the extreme right column; rows 1, 4, 6, 7 and 10 meet Criterion A of a ‘major depressive episode’ and have a diagnosis value of 1 (whereas rows not meeting Criterion A have a value of 0)

Although DSM-5 is widely used, it is inevitable that the criteria of diagnoses will change. However, because of the lack of a rationally valid method of storing data of DSM-5, in the current state, almost all results of past diagnoses are predicted to be incompatible with future upgrades such as a potential DSM-6. Additionally, the mere storing of data may exponentially increase and become infeasible; therefore, an efficient paradigm for storing data is considered necessary. To address this issue, it is intended that a potential profile based on symmetry via an abstract algebra will be applied in line with our previous reports [9, 10]. This study does not propose changes to the criteria, and therefore does not make a comparison of the effectiveness of existing and future criteria. Instead, it introduces a framework with which to manage changes in criteria.

## Methods

For the demonstration of our concept, the nine symptoms in Criterion A for a ‘major depressive episode’ of a ‘major depressive disorder’ in DSM-5 are described in a vector-like form as Cartesian products for modulo-2 (Z_2_^×n^; n: natural number; indicating the presence or absence of each symptom) or −7 (Z_7_^×n^; indicating the severity of each symptom) arithmetic, mainly addition. A novel concept for a value of zero being classified as explicit or implicit is then introduced to establish a unique set including the above vectors. For these vectors, the meanings of operators are given such that the operators not only indicate the diagnosis of patients and the severity of each symptom but also express the order for changes in them. Then, by the combination of Z_2_^×n^ and Z_7_^×n^, a method of transforming an original assessment of the severity of each symptom into updated/modified scores/scales is illustrated using inner products such as (Z_2_^×n^) · (Z_7_^×n^). Additionally, a multi-focal use as in (Z_2_^×M^)^×n^ is illustrated to express optional orders for psychiatrists.

### 1. Composition of modulo-2 arithmetic diagnosis and modulo-7 arithmetic scoring for a ‘major depressive episode’ in DSM-5

We first consider the DSM-5 system, focusing on Criterion A for a ‘major depressive episode’ as an example, and compose modulo-2 and −7 arithmetic (mainly addition). We consider the following scenario. We regard the binominal diagnosis system of DSM-5 as a two-point ordinal scale rated by a value {0 or 1} from the classification of S. S. Stevens [13]. In general, an ordinal scale does not always contain 0 as a score (e.g., {1, 2, 3, 4, 5}); however, to compose modulo addition, we suggest an ordinal rating scale containing 0 (e.g., {0, 1, 2, 3, 4}) (which we call a modular scale) [14]. By assigning the value 1 to the presence of a certain item of DSM-5 and the value 0 to absence, psychiatric disease states can be expressed with a base-2 system, e.g., Z_2_ = {0, 1}. As previously mentioned, Criterion A for a ‘major depressive episode’ consists of nine specific symptoms that have binominal scores for diagnosis. For simplification, if a certain patient is evaluated by a psychiatrist in clinical examination, whether he or she is categorized as having a ‘major depressive episode’ or not, the rating for that criterion is possible. The following results are obtained for the diagnosis of a ‘major depressive episode’.

Thereby, using the j-th assessment for the i-th symptom denoted a_(j)i_ (={0 or 1}) (item number i = 1, 2, 3,…, n) (j = 1, 2, 3,…), the state of Criterion A can be described in vector form adding ‘mod 2’ after the vector to refer to the modulo arithmetic:

1 $$ {\mathrm{A}}_{\mathrm{j}}=\left[{\mathrm{a}}_{\left(\mathrm{j}\right)1}\left|{\mathrm{a}}_{\left(\mathrm{j}\right)2}\right|{\mathrm{a}}_{\left(\mathrm{j}\right)3}\left|\dots \right|{\mathrm{a}}_{\left(\mathrm{j}\right)\mathrm{i}}\left|\dots \right|{\mathrm{a}}_{\left(\mathrm{j}\right)\mathrm{n}-1}\Big|{\mathrm{a}}_{\left(\mathrm{j}\right)\mathrm{n}}\right]\ \left( \mod\ 2\right)\left(\mathrm{n}:\ \mathrm{n}\mathrm{umber}\ \mathrm{of}\ \mathrm{practical}\ \mathrm{symptoms};\ \mathrm{n} = 9\ \mathrm{in}\ \mathrm{this}\ \mathrm{case}\right). $$

Hence, a 9-fold Cartesian product Z_2_ × Z_2_ × … × Z_2_ = Z_2_^×n^ (*n* = 9) belonging to a single modulo group/ring/field A = {A_j_ | A_j_ ∈ Z_2_^×n^} (*n* = 9) can be composed. Examples are presented in Table 1, and a compressed description is given in Table 2.

**Table 2:** Compressed expression of Table 1

Count		State	Row/Session		Abbreviation	Episode
3	×	A[110110101]	(4,7,10)	(mod 2)	3∙A_110110101_ (4,7,10)	3
2	×	A[011100001]	(5,9)	(mod 2)	2∙A_011100001_ (5,9)	0
2	×	A[010010011]	(2,8)	(mod 2)	2∙A_010010011_ (2,8)	0
1	×	A[111011011]	(1)	(mod 2)	A_111011011_ (1)	1
1	×	A[111011001]	(6)	(mod 2)	A_111011001_ (6)	1
1	×	A[000100010]	(3)	(mod 2)	A_000100010_ (3)	0
	Sum[5|9|4|6|7|2|3|4|9] (non-modular)					5
	Mean[0.5|0.9|0.4|0.6|0.7|0.2|0.3|0.4|0.9] (non-modular)					0.5

The second column from the right gives the compressed forms of the model, which can be used independently; here the rows in Table 1 that have the same series of numerals are simplified as a single row. The numerals immediately before ‘A_×××××××××_’ are the counts of patients who have the same series of assessments, and the rows are arranged in the descending order of the counts. For the same number of counts, assessments are listed in descending order of their base-2 notation (e.g., 110110101). The numerals in parentheses (e.g., (5,9)) indicate the original row numbers in Table 1. The diagnosis of whether the patient meets Criterion A of a ‘major depressive episode’ (denoted 1) or not denoted 0) is given in the extreme right column. In the table, the number of rows does not exceed 2^×9^, according to the characteristics of the group/ring/field Z_2_^×9^

Additionally, DSM-5 provides optional scales for assessment: the Self-rated Level 1 Cross-cutting Symptom Measure—Adult (13 items) and Parent/Guardian-rated Level 1 Cross-cutting Symptom Measure—Child Age 6–17 (12 items) where the set of scores is {0 (non), 1 (slight), 2 (mild), 3 (moderate), 4 (severe)}; the Clinician-rated Dimension of Psychosis Symptom Severity (eight items) with scores {0, 1, 2, 3, 4}; and the World Health Organization Disability Assessment Schedule 2.0 (WHODAS) (36 items) with scores {1, 2, 3, 4, 5}. With reference to these optional scales, items of Criterion A for a ‘major depressive episode’ in DSM-5 could be scored in more detail, e.g., using a five-point scale instead of a two-point scale. In particular, the use of a seven-point scale such as {0, 1, 2, 3, 4, 5, 6} could be advantageous. Naturally, the five-point and seven-point scales could obey modulo-5 and −7 arithmetic respectively. We consider that a p-grading scale (where p is a prime number) in accordance with the specific case is preferable. Because some common psychiatric evaluation scales, such as the Brief Psychiatric Rating Scale (BPRS) [15], the Positive and Negative Syndrome Scale (PANSS) for schizophrenia [16], the Montgomery–Åsberg Depression Rating Scale (MADRS) for depression [17] and the Clinical Global Impression of Severity for psychiatric disease [18], have seven grades, we consider modulo-7 arithmetic (especially addition) in the following. If the j-th assessment for the same patients combining a seven-point score that expresses the severity for the i-th symptom of Criterion A denoted ‹a_(j)i_› (={0, 1, 2, 3, 4, 5, 6}) is written as ‹A_j_›, (j = 1, 2, 3,…) (item number, i = 1, 2, 3,…, n), we then write

2 $$ \mbox{\fontencoding{T1}\fontfamily{cmr}\selectfont\char"0E} {\mathrm{A}}_{\mathrm{j}}\mbox{\fontencoding{T1}\fontfamily{cmr}\selectfont\char"0F} =\left[\mbox{\fontencoding{T1}\fontfamily{cmr}\selectfont\char"0E} {\mathrm{a}}_{\left(\mathrm{j}\right)1}\mbox{\fontencoding{T1}\fontfamily{cmr}\selectfont\char"0F} \left|\mbox{\fontencoding{T1}\fontfamily{cmr}\selectfont\char"0E} {\mathrm{a}}_{\left(\mathrm{j}\right)2}\mbox{\fontencoding{T1}\fontfamily{cmr}\selectfont\char"0F} \right|\mbox{\fontencoding{T1}\fontfamily{cmr}\selectfont\char"0E} {\mathrm{a}}_{\left(\mathrm{j}\right)3}\mbox{\fontencoding{T1}\fontfamily{cmr}\selectfont\char"0F} \Big|\dots \left|\mbox{\fontencoding{T1}\fontfamily{cmr}\selectfont\char"0E} {\mathrm{a}}_{\left(\mathrm{j}\right)\mathrm{i}}\mbox{\fontencoding{T1}\fontfamily{cmr}\selectfont\char"0F} \right|\dots \left|\mbox{\fontencoding{T1}\fontfamily{cmr}\selectfont\char"0E} {\mathrm{a}}_{\left(\mathrm{j}\right)\left(\mathrm{n}-1\right)}\mbox{\fontencoding{T1}\fontfamily{cmr}\selectfont\char"0F} \right|\mbox{\fontencoding{T1}\fontfamily{cmr}\selectfont\char"0E} {\mathrm{a}}_{\left(\mathrm{j}\right)\mathrm{n}}\mbox{\fontencoding{T1}\fontfamily{cmr}\selectfont\char"0F} \right]\ \left( \mod\ 7\right)\ \left(\mathrm{n} = 9\right) $$

(a patient’s state of severity in the j-th session on Criterion A).

In the same manner, a 9-fold Cartesian product Z_7_ × Z_7_ × … × Z_7_ = Z_7_^×n^ (*n* = 9) belonging to a single modulo group/ring/field ‹A› = {‹A_j_›|‹A_j_› ∈ Z_7_^×n^} (*n* = 9) can be defined. Examples are given in Table 3, and a compressed version is given in Table 4. The diagnosis for presence/absence of a ‘major depressive episode’ is presented in the extreme right columns of Tables 1–4. Note that the diagnosis for respective rows is identical between Tables 1 and 3 and between Tables 2 and 4.

**Table 3:** Presentation of examples of the severity assessment on Criterion A

Symptom number	1	2	3	4	5	6	7	8	9		Episode
row 1	2	5	3	0	6	2	0	4	1	(mod 7)	1
row 2	0	2	0	0	4	0	0	2	3	(mod 7)	0
row 3	0	0	0	5	0	0	0	3	0	(mod 7)	0
row 4	5	4	0	2	1	0	3	0	6	(mod 7)	1
row 5	0	6	4	1	0	0	0	0	2	(mod 7)	0
row 6	1	5	3	0	1	4	0	0	1	(mod 7)	1
row 7 (= row 4)	5	4	0	2	1	0	3	0	6	(mod 7)	1
row 8 (= row 2)	0	2	0	0	4	0	0	2	3	(mod 7)	0
row 9 (= row 5)	0	6	4	1	0	0	0	0	2	(mod 7)	0
row 10 (= row 4)	5	4	0	2	1	0	3	0	6	(mod 7)	1
Total sum	18	38	14	13	18	6	9	11	30		5
Average	1.8	3.8	1.4	1.3	1.8	0.6	0.9	1.1	3.0		0.5

Examples for symptoms 1–9 in Criterion A composed of ‘0, 1, 2,…, 6’ are presented. Rows 7 and 10 are equivalent to row 4; i.e., row 4 = row 7 = row 10. Additionally, row 2 = row 8 and row 5 = row 9. The table gives the order of effectiveness of symptoms 1–9 in Criterion A. Similar to the case of diagnosis (Tables 1 and 2), all symptoms 1–9 in Criterion A are effective, which is expressed by A_all(1–9)_ (= A_(0→all(1–9))_) = [1_1_|1_2_|1_3_|1_4_|1_5_|1_6_|1_7_|1_8_|1_9_||**0**_**10**_|**0**_**11**_|**0**_**12**_|…] (mod 2). Note that A_all(1-n)_ (in this regard, n = 9) acts as an identity for an inner product; ‹A_j_› ∙ A_all(1-n)_ = A_all(1-n)_ ∙ ‹A_j_› = ‹A_j_› (mod 7) (*n* = 9). Additionally, ‹A_j_› could be regarded as an operator that yields ‹A_j_› (= ‹A_(0→j)_›) itself by acting on ‹A_0_›; ‹A_0_› *‹A_j_› = ‹A_0_› *‹A_(0→j)_› = ‹A_j_› (mod 7), where ‹A_0_› is an identity (unrated or completely healthy) state ‹A_0_› = [0_1_|0_2_|0_3_|0_4_|0_5_|0_6_|0_7_|0_8_|0_9_||**0**_**10**_|**0**_**11**_|**0**_**12**_|…] (mod 7)

**Table 4:** Compressed expression of Table 3

Count		State	Row/Session		Abbreviation	Episode
3	×	A[540210306]	(4,7,10)	(mod 7)	3∙‹A›_540210306_ (4,7,10)	3
2	×	A[064100002]	(5,9)	(mod 7)	2∙‹A›_064100002_ (5,9)	0
2	×	A[020040023]	(2,8)	(mod 7)	2∙‹A›_020040023_ (2,8)	0
1	×	A[253062041]	(1)	(mod 7)	‹A›_253062041_ (1)	1
1	×	A[153014001]	(6)	(mod 7)	‹A›_153014001_ (6)	1
1	×	A[000500030]	(3)	(mod 7)	‹A›_000500030_ (3)	0
	‹Sum›[18|38|14|13|18|6|9|11|30] (non-modular)					5
	‹Mean›[1.8|3.8|1.4|1.3|1.8|0.6|0.9|1.1|3.0] (non-modular)					0.5

The rows in Table 3 that have the same series of numerals are simplified as a single row. The numerals immediately before ‘‹A›_×××××××××_’ are the counts of patients who have the same series of assessments, and the rows are arranged in descending order of the number of counts. For the same number of counts, rows are listed in descending order of their base-7 notation (e.g., 540210306). Numerals in parentheses (e.g., (2,8)) indicate the original row numbers in Table 3. In the table, the number of rows does not exceed 7^×9^, according to the characteristics of the group/ring/field Z_7_^×9^. The right column is considered the minimized form of the model and can be used independently

For a more concrete demonstration, we consider an example of a pair of assessments in the j-th session:

3 $$ {\mathrm{A}}_{\mathrm{j}}=\left[{1}_1\left|{1}_2\right|{1}_3\left|{0}_4\right|{1}_5\left|{1}_6\right|{0}_7\left|{1}_8\right|{1}_9\right]\ \left( \mod\ 2\right), $$

4 $$ \mbox{\fontencoding{T1}\fontfamily{cmr}\selectfont\char"0E} {\mathrm{A}}_{\mathrm{j}}\mbox{\fontencoding{T1}\fontfamily{cmr}\selectfont\char"0F} =\left[{2}_1\left|{5}_2\right|{3}_3\left|{0}_4\right|{6}_5\left|{2}_6\right|{0}_7\left|{4}_8\right|{1}_9\right]\ \left( \mod\ 7\right), $$

where the indexes refer to the order of the components. Examples are presented in Table 1 including formula (3) in the first row; a compressed version is given in Table 2. Similarly, Table 3 gives examples including formula (4) in the first row; a compressed version is given in Table 4. Note that the numbers 2 and 7 being primes allows modulo arithmetic operation (addition, subtraction, multiplication and division). Additionally, in (3) and (4), the component 0 should always appear at the same moment because (4) is a series of severity that provides detail to (3). This can be expressed using the inner product ‘∙’ as

5 $$ {\mathrm{A}}_{\mathrm{j}}\cdot \mbox{\fontencoding{T1}\fontfamily{cmr}\selectfont\char"0E} {\mathrm{A}}_{\mathrm{j}} \mbox{\fontencoding{T1}\fontfamily{cmr}\selectfont\char"0F} \left( \mod\ 7\right) = \mbox{\fontencoding{T1}\fontfamily{cmr}\selectfont\char"0E} {\mathrm{A}}_{\mathrm{j}}\mbox{\fontencoding{T1}\fontfamily{cmr}\selectfont\char"0F} \cdot {\mathrm{A}}_{\mathrm{j}}\ \left( \mod\ 7\right) = \mbox{\fontencoding{T1}\fontfamily{cmr}\selectfont\char"0E} {\mathrm{A}}_{\mathrm{j}} \mbox{\fontencoding{T1}\fontfamily{cmr}\selectfont\char"0F} \left( \mod\ 7\right). $$

As an example, using (3) and (4), A_j_ ∙ ‹A_j_› (mod 7)

$$ \begin{array}{l}=\left[{1}_1\cdot {2}_1\left|{1}_2\cdot {5}_2\right|{1}_3\cdot {3}_3\left|{0}_4\cdot {0}_4\right|{1}_5\cdot {6}_5\left|{1}_6\cdot {2}_6\right|{0}_7\cdot {0}_7\left|{1}_8\cdot {4}_8\right|{1}_9\cdot {1}_9\right]\ \left( \mod\ 7\right),\\ {}=\left[{2}_1\left|{5}_2\right|{3}_3\left|{0}_4\right|{6}_5\left|{2}_6\right|{0}_7\left|{4}_8\right|{1}_9\right]\ \left( \mod\ 7\right).\end{array} $$

Simple multiplications are performed between the components having the same index in the two vectors ignoring modular arithmetic. Results should then be reinterpreted using modulo-7 arithmetic.

However, the second ‘=’ in equation (5) refers not to an identity but to a specific relationship according to the definition of symptoms in DSM-5. Indeed, the combination of different numbers for each component such as 0 (mod 2)/5 (mod 7) or 1 (mod 2)/0 (mod 7) is permissible within Z_2_ × Z_7_.

### 2. Introduction of an optional concept of ‘0’ for upgrading criterion/scales via incorporation/deletion of symptoms

When we upgrade the criterion for a certain disease state or devise a scale or subscale, it is often necessary to incorporate or delete specific items so that items are more appropriate. For example, we might incorporate ‘fear’ as a new symptom in Criterion A for a ‘major depressive episode’ between the fourth and fifth items in […|a_(j)4_||a_(j)5_|…], and delete the sixth item ‘fatigue or loss of energy every day’ from the original criterion of DM-5. To treat these cases using the same modulo arithmetic, we introduce a novel rule for the value 0. We classify the value of 0 as being explicit or implicit. The explicit 0 is an ordinal 0 written as a numeral and accompanied by an index such as in the case of 0_5_ in the vector […|a_(j)4_|**0**_**5**_|a_(j)6_|…]. The implicit 0 is not written as a numeral and it is postulated that we can find it freely in any interval between neighboring components, e.g., between the fourth and fifth items in […|a_(j)4_**()()()**…a_(j)5_|…]. The conversion between an explicit 0 and implicit 0 as in **0**_**5**_**↔()** is permitted under the condition that all changes are recognized. A similar idea was introduced in our previous report [19]. Practical details are given in sections 3, 4 and 5.

### 3. Examples of the selection of symptoms for Criterion A based on modulo-2 arithmetic

In composing a group (potentially a ring or field), there are an infinite number of explicit 0 s and the trailing series of 0 s are implicitly implied; i.e., (3) and (4) are rewritten as

6 $$ {\mathrm{A}}_{\mathrm{j}}=\left[{1}_1\left|{1}_2\right|{1}_3\left|{0}_4\right|{1}_5\left|{1}_6\right|{0}_7\left|{1}_8\right|{1}_9\left|\left|{\mathbf{0}}_{\mathbf{10}}\right|{\mathbf{0}}_{\mathbf{11}}\right|{\mathbf{0}}_{\mathbf{12}}\Big|\dots \right]\ \left( \mod\ 2\right) $$

(a diagnosis in the j-th session on Criterion A for a certain patient),

7 $$ \mbox{\fontencoding{T1}\fontfamily{cmr}\selectfont\char"0E} {\mathrm{A}}_{\mathrm{j}}\mbox{\fontencoding{T1}\fontfamily{cmr}\selectfont\char"0F} =\left[{2}_1\left|{5}_2\right|{3}_3\left|{0}_4\right|{6}_5\left|{2}_6\right|{0}_7\left|{4}_8\right|{1}_9\left|\left|{\mathbf{0}}_{\mathbf{10}}\right|{\mathbf{0}}_{\mathbf{11}}\right|{\mathbf{0}}_{\mathbf{12}}\Big|\dots \right]\ \left( \mod\ 7\right) $$

(a state of severity in the j-th session on Criterion A for a certain patient).

Note that (6) and (7) have dual meanings similar to the case for a positional vector: 1) an absolute state A_j_ expressing the presence or absence of specific symptoms for simple diagnosis; and 2) an operator that changes a completely healthy state within Criterion A denoted A_0_ to a disease state A_j_ by A_0_ * A_(0→j)_ = A_j_, where

8 $$ {\mathrm{A}}_0=\left[{0}_1\left|{0}_2\right|{0}_3\left|{0}_4\right|{0}_5\left|{0}_6\right|{0}_7\left|{0}_8\right|{0}_9\left|\left|{\mathbf{0}}_{\mathbf{10}}\right|{\mathbf{0}}_{\mathbf{11}}\right|{\mathbf{0}}_{\mathbf{12}}\Big|\dots \right]\ \left( \mod\ 2\right) $$

is the completely healthy state for item A. In the case of the latter meaning, the act of diagnosis is an operation A_j_ on A_0_, and the act of assessment of severity on Criterion A is an operation ‹A_j_› on ‹A_0_›.

In example (3), a new component for ‘fear’ is found between items 4 and 5 as an implicit 0; i.e.,

9 $$ {\mathrm{A}}_{\mathrm{j}}=\left[{1}_1\left|{1}_2\right|{1}_3\left|{0}_4\left(\ \right){1}_5\right|{1}_6\left|{0}_7\right|{1}_8\left|{1}_9\right|\left|{\mathbf{0}}_{\mathbf{10}}\right|{\mathbf{0}}_{\mathbf{11}}\left|{\mathbf{0}}_{\mathbf{12}}\right|\dots \right]\ \left( \mod\ 2\right). $$

The implicit 0 is then converted to an explicit 0:

10 $$ \begin{array}{l}{\dot{\mathrm{A}}}_{\mathrm{j}}=\left[{1}_1\left|{1}_2\right|{1}_3\left|{0}_4\left({\mathbf{0}}_{\mathbf{5}}\right){1}_6\right|{1}_7\left|{0}_8\right|{1}_9\left|{1}_{10}\right|\left|{\mathbf{0}}_{\mathbf{11}}\right|{\mathbf{0}}_{\mathbf{12}}\left|{\mathbf{0}}_{\mathbf{13}}\right|\dots \right]\ \left( \mod\ 2\right).\\ {}\end{array} $$

Expression (10) implies an absence denoted 0_5_ (fifth symptom; fear), and index numbers after the index 4 increase by 1 because of the emergence of the item 0_5_ in (10). Then, if we express the fifth symptom (fear) with a presence denoted 1_5_, the following calculation provides this state.

$$ \mathrm{Through}\kern0.5em {\dot{\mathrm{A}}}_{\mathrm{x}} = \left[{0}_1\left|{0}_2\right|{0}_3\left|{0}_4\right|{\mathbf{1}}_{\mathbf{5}}\left|{0}_6\right|{0}_7\left|{0}_8\right|{0}_9\left|{1}_{10}\right|\left|{\mathbf{0}}_{\mathbf{1}\mathbf{1}}\right|{\mathbf{0}}_{\mathbf{1}\mathbf{2}}\left|{\mathbf{0}}_{\mathbf{1}\mathbf{3}}\right|\dots \right]\ \mathrm{acting}\ \mathrm{on}\kern0.5em {\dot{\mathrm{A}}}_{\mathrm{j}}, $$

modulo-2 arithmetic denotes collectiveness by † (whereas it denotes addition (including subtraction) by *, multiplication by × and division by /). In a simple example of modulo-2 addition,

11 $$ {\dot{\mathrm{A}}}_{\mathrm{j}}*\ {\dot{\mathrm{A}}}_{\mathrm{x}} = {\dot{\mathrm{A}}}_{\mathrm{j}} + {\dot{\mathrm{A}}}_{\mathrm{x}}\left( \mod\ 2\right)=\left[{1}_1+{0}_1\left|{1}_2+{0}_2\right|{1}_3+{0}_3\left|{0}_4+{0}_4\right|{0}_5+{1}_5\left|{1}_6+{0}_6\right|{1}_7+{0}_7\left|{0}_8+{0}_8\right|{1}_9+{0}_9\left|{1}_{10}+{0}_{10}\right|\left|{\mathbf{0}}_{\mathbf{1}\mathbf{1}}+{\mathbf{0}}_{\mathbf{1}\mathbf{1}}\right|{\mathbf{0}}_{\mathbf{1}\mathbf{2}}+{\mathbf{0}}_{\mathbf{1}\mathbf{2}}\left|{\mathbf{0}}_{\mathbf{1}\mathbf{3}}+{\mathbf{0}}_{\mathbf{1}\mathbf{3}}\right| \dots \left]\left( \mod\ 2\right) = \right[{1}_1\left|{1}_2\right|{1}_3\left|{0}_4\left({\mathbf{1}}_{\mathbf{5}}\right){1}_6\right|{1}_7\left|{0}_8\right|{1}_9\left|{1}_{10}\right|\left|{\mathbf{0}}_{\mathbf{1}\mathbf{1}}\right|{\mathbf{0}}_{\mathbf{1}\mathbf{2}}\left|{\mathbf{0}}_{\mathbf{1}\mathbf{3}}\right|\dots \right]\ \left( \mod\ 2\right)={\hat{\mathrm{A}}}_{\mathrm{k}}. $$

Moreover, to delete the eighth item ‘diminished ability to think or concentrate, or indecisiveness, nearly every day’ from the original Criterion A (i.e., the ninth component in (11)), first, the item 1_9_ in Â_k_ should be changed to 0 through the action of an operator with modulo-2 arithmetic (e.g., addition denoted *) Â_y_ on Â_k_:

12 $$ {\hat{\mathrm{A}}}_{\mathrm{y}}=\left[{0}_1\left|{0}_2\right|{0}_3\left|{0}_4\right|{0}_5\left|{0}_6\right|{0}_7\left|{0}_8\right|+{\mathbf{1}}_{\mathbf{9}}\left|{0}_{10}\right|\left|{\mathbf{0}}_{\mathbf{1}\mathbf{1}}\right|{\mathbf{0}}_{\mathbf{1}\mathbf{2}}\left|{\mathbf{0}}_{\mathbf{1}\mathbf{3}}\right|\dots \right]\ \left( \mod\ 2\right),\;{\hat{\mathrm{A}}}_{\mathrm{k}}*\ {\hat{\mathrm{A}}}_{\mathrm{y}} = {\hat{\mathrm{A}}}_{\mathrm{k}} + {\hat{\mathrm{A}}}_{\mathrm{y}}\left( \mod\ 2\right)=\left[{1}_1\left|{1}_2\right|{1}_3\left|{0}_4\right|{1}_5\left|{1}_6\right|{1}_7\left|{0}_8\right|{\mathbf{0}}_{\mathbf{9}}\left|{1}_{10}\right|\left|{\mathbf{0}}_{\mathbf{1}\mathbf{1}}\right|{\mathbf{0}}_{\mathbf{1}\mathbf{2}}\left|{\mathbf{0}}_{\mathbf{1}\mathbf{3}}\right|\dots \right]\left( \mod\ 2\right)={\tilde{\mathrm{A}}}_{\mathrm{m}} $$

(see Appendix A).

Next, by changing the ninth component from an explicit 0 to an implicit 0, the final state is obtained as

13 $$ {\tilde{\mathrm{A}}}_{\mathrm{m}}\to\ {\mathring{\mathrm{A}}}_{\mathrm{m}}=\left[{1}_1\left|{1}_2\right|{1}_3\left|{0}_4\right|{1}_5\left|{1}_6\right|{1}_7\left|{0}_8\left(\ \right){1}_9\right|{\mathbf{0}}_{\mathbf{10}}\left|\left|{\mathbf{0}}_{\mathbf{11}}\right|{\mathbf{0}}_{\mathbf{12}}\right|\dots \right]\ \left( \mod\ 2\right) $$

14 $$ =\left[{1}_1\left|{1}_2\right|{1}_3\left|{0}_4\right|{1}_5\left|{1}_6\right|{1}_7\left|{0}_8\right|{1}_9\left|\left|{\mathbf{0}}_{\mathbf{10}}\right|{\mathbf{0}}_{\mathbf{11}}\right|{\mathbf{0}}_{\mathbf{12}}\Big|\dots \right]\ \left( \mod\ 2\right). $$

The index numbers after the index 9 reduce by 1 because of the deletion of the item 0_9_ in (12). Simple illustrations and a compressed version are presented in Tables 5 and 6, where the extreme right column gives the presence/absence of a ‘major depressive episode’ under the same diagnostic condition as in Tables 1–4.

**Table 5:** Presentation of examples after the modification of Criterion A

Symptom number	1	2	3	4	5(fear)	6	7	8	9		Episode
row 1	1	1	1	0	1	1	1	0	1	(mod 2)	1
row 2	0	1	0	0	0	1	0	0	1	(mod 2)	0
row 3	0	0	0	1	1	0	0	0	0	(mod 2)	0
row 4	1	1	0	1	1	1	0	1	1	(mod 2)	1
1row 5	0	1	1	1	0	0	0	0	1	(mod 2)	0
row 6 (= row 1)	1	1	1	0	1	1	1	0	1	(mod 2)	1
row 7	1	1	0	1	1	1	0	1	1	(mod 2)	1
row 8 (= row 2)	0	1	0	0	0	1	0	0	1	(mod 2)	0
0row 9 (= row 5)	0	1	1	1	1	0	0	0	1	(mod 2)	1
row 10 (= row 4)	1	1	0	1	1	1	0	1	1	(mod 2)	1
Total sum	5	9	4	6	7	7	2	3	9		6
Average	0.5	0.9	0.4	0.6	0.7	0.7	0.2	0.3	0.9		0.6

A new item ‘fear’ was incorporated between symptoms 4 and 5, and the ninth symptom ‘diminished ability’ (eighth symptom of the original Criterion A) was deleted. The index numbers 4–7 were then increased by 1 to the numbers 5–8, and the index numbers 10, 11,… were reduced by 1 to the numbers 9, 10,…

**Table 6:** Compressed expression of Table 5

Counts		States	Row/Session		Abbreviation	Episode
3	×	A[110111011]	(4,7,10)	(mod 2)	3∙A_110111011_ (4,7,10)	3
2	×	A[111011101]	(1,6)	(mod 2)	2∙A_111011101_ (1,6)	2
2	×	A[010001001]	(2,8)	(mod 2)	2∙A_010001001_ (2,8)	0
1	×	A[011110001]	(9)	(mod 2)	A_011110001_ (9)	1
1	×	A[011100001]	(5)	(mod 2)	A_011100001_ (5)	0
1	×	A[000110000]	(3)	(mod 2)	A_000110000_ (3)	0
	Sum[5|9|4|6|7|7|2|3|9] (non-modular)					6
	Mean[0.5|0.9|0.4|0.6|0.7|0.7|0.2|0.3|0.9] (non-modular)					0.6

The examples of Figure 5 are presented in compressed form as for Tables 2 and 4

If supplemented, incorporation and deletion are also applicable within ordinal modulo arithmetic (e.g., addition) because all procedures in the above manipulation are accompanied by operators (see Appendix B for details).

However, step-by-step manipulation can be troublesome. All incorporations and deletions via conversion between implicit 0 s and explicit 0 s such as **0**_**i**_**↔()** are accompanied with a modulo arithmetic operation. We can thus perform this conversion freely with less manipulation employing modulo arithmetic.

As a further example, multiple criteria/scales can be combined as follows.

Let A_a_ = [1_1_|1_2_|0_3_|0_4_|1_5_||**0**_**6**_|**0**_**7**_|**0**_**8**_|…] (mod 2) (where there are five effective symptoms of a certain criterion) and A_b_ = [1_1_|0_2_|1_3_||**0**_**4**_|**0**_**5**_|**0**_**6**_|…] (mod 2) (where there are three effective symptoms).

So that the index numbers are the same, 0 s are incorporated:

$$ {\mathrm{A}}_{\mathrm{a}}\to {\mathrm{A}}_{\mathrm{a}1}=\left[{1}_1\left|{1}_2\right|{0}_3\left|{0}_4\right|{1}_5\left|{\mathbf{0}}_{\mathbf{6}}\right|{\mathbf{0}}_{\mathbf{7}}\left|{\mathbf{0}}_{\mathbf{8}}\right|\left|{\mathbf{0}}_{\mathbf{9}}\right|{\mathbf{0}}_{\mathbf{1}0}\left|{\mathbf{0}}_{\mathbf{1}1}\right|{\mathbf{0}}_{\mathbf{1}\mathbf{2}}\Big|\dots \right]\ \left( \mod\ 2\right), $$

$$ {\mathrm{A}}_{\mathrm{b}}\to {\mathrm{A}}_{\mathrm{b}1}=\left[{\mathbf{0}}_{\mathbf{1}}\left|{\mathbf{0}}_{\mathbf{2}}\right|{\mathbf{0}}_{\mathbf{3}}\left|{\mathbf{0}}_{\mathbf{4}}\right|{\mathbf{0}}_{\mathbf{5}}\left|{1}_{1+5}\right|{0}_{2+5}\left|{1}_{3+5}\left|\left|{\mathbf{0}}_{\mathbf{4}+\mathbf{5}}\right|{\mathbf{0}}_{\mathbf{5}+\mathbf{5}}\right|{\mathbf{0}}_{\mathbf{6}+\mathbf{5}}\right|\dots \right]\ \left( \mod\ 2\right). $$

The assessment over combined criterion

15 $$ {\mathrm{A}}_{\mathrm{c}} = \left\{{\mathrm{A}}_{\mathrm{a}},\ {\mathrm{A}}_{\mathrm{b}}\right\}\ \left( \mod\ 2\right) = {\mathrm{A}}_{\mathrm{a}1}*\ {\mathrm{A}}_{\mathrm{b}1}\left( \mod\ 2\right) = {\mathrm{A}}_{\mathrm{a}1} + {\mathrm{A}}_{\mathrm{b}1}\left( \mod\ 2\right) = \left[{\mathrm{A}}_{\mathrm{a}}\left|{\mathrm{A}}_{\mathrm{b}}\right| \dots \left]\left( \mod\ 2\right) = \right[{1}_1\left|{1}_2\right|{0}_3\left|{0}_4\right|{1}_5\left|{1}_6\right|{0}_7\left|{1}_8\left|\left|{\mathbf{0}}_{\mathbf{9}}\right|{\mathbf{0}}_{\mathbf{10}}\right|{\mathbf{0}}_{\mathbf{11}}\right|\dots \right]\ \left( \mod\ 2\right) $$

is obtained. Naturally, other combinations such as

16 $$ {\mathrm{A}}_{\mathrm{e}} = \left\{{\mathrm{A}}_{\mathrm{c}},\ {\mathrm{A}}_{\mathrm{d}}\right\}\ \left( \mod\ 2\right) = \left[{\mathrm{A}}_{\mathrm{c}}\left|{\mathrm{A}}_{\mathrm{d}}\right|\dots \right]\ \left( \mod\ 2\right) $$

can be composed freely. (A demonstration is presented in Appendix C.)

Here A_a_ expresses disease I, A_b_ expresses diseases I and II, A_c_ expresses diseases I, II and III, A_d_ expresses diseases I − IV, and A_e_ expresses diseases I − V.

Additionally, various types of incorporation, deletion and combinations are considered possible. Furthermore, any or all criteria of DSM-5 can be brought into line in a unique vector belonging to a single set A = {A_j_ | A_j_ ∈ Z_2_^×∞^}. This approach is similar to a coding method that we previously reported for indels of deoxyribonucleic acid sequences [19]. Evidently, modulo-2 (or −7) multiplication and division can also be performed although their optimal applications are to be explored in future studies.

### 4. Examples of tracing the scores of seven-point severity in accordance with changes to Criterion A

For modulo-7 arithmetic, we incorporate the new item ‘fear’ rated with a score of 3 between the fourth and fifth items of ‹A_j_›, and delete the eighth item ‘diminished ability to think or concentrate, or indecisiveness, nearly every day’ from the original Criterion A. In the case of incorporation, first, the implicit 0 is found between the fourth and fifth items:

17 $$ \mbox{\fontencoding{T1}\fontfamily{cmr}\selectfont\char"0E} {\mathrm{A}}_{\mathrm{j}}\mbox{\fontencoding{T1}\fontfamily{cmr}\selectfont\char"0F} =\left[{2}_1\left|{5}_2\right|{3}_3\left|{0}_4\left(\ \right){6}_5\right|{2}_6\left|{0}_7\right|{4}_8\left|{1}_9\right|\left|{\mathbf{0}}_{\mathbf{10}}\right|{\mathbf{0}}_{\mathbf{11}}\left|{\mathbf{0}}_{\mathbf{12}}\right|\dots \right]\ \left( \mod\ 7\right). $$

Next, the implicit 0 is changed to an explicit 0:

18 $$ \mbox{\fontencoding{T1}\fontfamily{cmr}\selectfont\char"0E} {\dot{\mathrm{A}}}_{\mathrm{j}}\mbox{\fontencoding{T1}\fontfamily{cmr}\selectfont\char"0F} =\left[{2}_1\left|{5}_2\right|{3}_3\left|{0}_4\left({\mathbf{0}}_{\mathbf{5}}\right){6}_6\right|{2}_7\left|{0}_8\right|{4}_9\left|{1}_{10}\right|\left|{\mathbf{0}}_{\mathbf{11}}\right|{\mathbf{0}}_{\mathbf{12}}\left|{\mathbf{0}}_{\mathbf{13}}\right|\dots \right]\ \left( \mod\ 7\right). $$

$$ \mathrm{From}\mbox{\fontencoding{T1}\fontfamily{cmr}\selectfont\char"0E} {\dot{\mathrm{A}}}_{\mathrm{j}}\mbox{\fontencoding{T1}\fontfamily{cmr}\selectfont\char"0F}, \mathrm{b}\mathrm{y}\ \mathrm{the}\ \mathrm{a}\mathrm{ction}\ \mathrm{of}\ `\mbox{\fontencoding{T1}\fontfamily{cmr}\selectfont\char"0E} {\dot{\mathrm{A}}}_{\mathrm{x}} \mbox{\fontencoding{T1}\fontfamily{cmr}\selectfont\char"0F} = \left[{0}_1\left|{0}_2\right|{0}_3\left|{0}_4\right|{\mathbf{3}}_{\mathbf{5}}\left|{0}_6\right|{0}_7\left|{0}_8\right|{0}_9\left|{0}_{10}\right|\left|{\mathbf{0}}_{\mathbf{11}}\right|{\mathbf{0}}_{\mathbf{12}}\left|{\mathbf{0}}_{\mathbf{13}}\right|\dots \right]',\mbox{\fontencoding{T1}\fontfamily{cmr}\selectfont\char"0E} {\hat{\mathrm{A}}}_{\mathrm{k}}\mbox{\fontencoding{T1}\fontfamily{cmr}\selectfont\char"0F} \mathrm{is}\ \mathrm{obtained}\ \mathrm{a}\mathrm{s} $$

19 $$ \mbox{\fontencoding{T1}\fontfamily{cmr}\selectfont\char"0E} {\hat{\mathrm{A}}}_{\mathrm{k}}\mbox{\fontencoding{T1}\fontfamily{cmr}\selectfont\char"0F} =\mbox{\fontencoding{T1}\fontfamily{cmr}\selectfont\char"0E} {\dot{\mathrm{A}}}_{\mathrm{j}}\mbox{\fontencoding{T1}\fontfamily{cmr}\selectfont\char"0F} *\mbox{\fontencoding{T1}\fontfamily{cmr}\selectfont\char"0E} {\dot{\mathrm{A}}}_{\mathrm{x}}\mbox{\fontencoding{T1}\fontfamily{cmr}\selectfont\char"0F} =\mbox{\fontencoding{T1}\fontfamily{cmr}\selectfont\char"0E} {\dot{\mathrm{A}}}_{\mathrm{j}}\mbox{\fontencoding{T1}\fontfamily{cmr}\selectfont\char"0F} +\mbox{\fontencoding{T1}\fontfamily{cmr}\selectfont\char"0E} {\dot{\mathrm{A}}}_{\mathrm{x}}\mbox{\fontencoding{T1}\fontfamily{cmr}\selectfont\char"0F} =\left[{2}_1\left|{5}_2\right|{3}_3\left|{0}_4\left({\mathbf{0}}_{\mathbf{5}}\right){6}_6\right|{2}_7\left|{0}_8\right|{4}_9\left|{1}_{10}\right|\left|{\mathbf{0}}_{\mathbf{11}}\right|{\mathbf{0}}_{\mathbf{12}}\left|{\mathbf{0}}_{\mathbf{13}}\right|\dots \right] + \left[{0}_1\left|{0}_2\right|{0}_3\left|{0}_4\right|{\mathbf{3}}_{\mathbf{5}}\left|{0}_6\right|{0}_7\left|{0}_8\right|{0}_9\left|{0}_{10}\right|\left|{\mathbf{0}}_{\mathbf{11}}\right|{\mathbf{0}}_{\mathbf{12}}\left|{\mathbf{0}}_{\mathbf{13}}\right|\dots \right]\ \left( \mod\ 7\right) = \left[{2}_1\left|{5}_2\right|{3}_3\left|{0}_4\left({\mathbf{3}}_{\mathbf{5}}\right){6}_6\right|{2}_7\left|{0}_8\right|{4}_9\left|{1}_{10}\right|\left|{\mathbf{0}}_{\mathbf{11}}\right|{\mathbf{0}}_{\mathbf{12}}\left|{\mathbf{0}}_{\mathbf{13}}\right|\dots \right]\ \left( \mod\ 7\right). $$

Then, to delete the eighth item ‘diminished ability to think or concentrate, or indecisiveness, nearly every day’ from the original Criterion A, first, the ninth component of (19), 4_9_, is changed to 0 through the action of an operator with modulo-7 arithmetic (e.g., addition denoted *) ‹Â_y_› on ‹Â_k_›:

20 $$ \begin{array}{l}\mbox{\fontencoding{T1}\fontfamily{cmr}\selectfont\char"0E} {\hat{\mathrm{A}}}_{\mathrm{y}}\mbox{\fontencoding{T1}\fontfamily{cmr}\selectfont\char"0F} = \left[{0}_1\left|{0}_2\right|{0}_3\left|{0}_4\right|{0}_5\left|{0}_6\right|{0}_7\left|{0}_8\right|{\mathbf{3}}_{\mathbf{9}}\left|{0}_{10}\right|\left|{\mathbf{0}}_{\mathbf{11}}\right|{\mathbf{0}}_{\mathbf{12}}\left|{\mathbf{0}}_{\mathbf{13}}\right|\dots \right]\ \left( \mod\ 7\right),\\ {}\mbox{\fontencoding{T1}\fontfamily{cmr}\selectfont\char"0E} {\hat{\mathrm{A}}}_{\mathrm{k}} \mbox{\fontencoding{T1}\fontfamily{cmr}\selectfont\char"0F} * \mbox{\fontencoding{T1}\fontfamily{cmr}\selectfont\char"0E} {\hat{\mathrm{A}}}_{\mathrm{y}} \mbox{\fontencoding{T1}\fontfamily{cmr}\selectfont\char"0F} = \mbox{\fontencoding{T1}\fontfamily{cmr}\selectfont\char"0E} {\hat{\mathrm{A}}}_{\mathrm{k}} \mbox{\fontencoding{T1}\fontfamily{cmr}\selectfont\char"0F} + \mbox{\fontencoding{T1}\fontfamily{cmr}\selectfont\char"0E} {\hat{\mathrm{A}}}_{\mathrm{y}} \mbox{\fontencoding{T1}\fontfamily{cmr}\selectfont\char"0F} = \mbox{\fontencoding{T1}\fontfamily{cmr}\selectfont\char"0E} {\tilde{\mathrm{A}}}_{\mathrm{m}}\mbox{\fontencoding{T1}\fontfamily{cmr}\selectfont\char"0F} .\end{array} $$

(see Appendix D for details)

$$ = \left[{2}_1\left|{5}_2\right|{3}_3\left|{0}_4\right|{3}_5\left|{6}_6\right|{2}_7\left|{0}_8\right|{\mathbf{0}}_{\mathbf{9}}\left|{1}_{10}\right|\left|{\mathbf{0}}_{\mathbf{11}}\right|{\mathbf{0}}_{\mathbf{12}}\left|{\mathbf{0}}_{\mathbf{13}}\right|\dots \right]\ \left( \mod\ 7\right). $$

The transformation ‹Ã_m_› → ‹Å_m_› is obtained with ‹Ã_z_›, where

21 $$ \mbox{\fontencoding{T1}\fontfamily{cmr}\selectfont\char"0E} {\tilde{\mathrm{A}}}_{\mathrm{z}}\mbox{\fontencoding{T1}\fontfamily{cmr}\selectfont\char"0F} =\left[{0}_1\left|{0}_2\right|{0}_3\left|{0}_4\right|{0}_5\left|{0}_6\right|{0}_7\left|{0}_8\right|{1}_9\left|{6}_{10}\right|\left|{\mathbf{0}}_{\mathbf{11}}\right|{\mathbf{0}}_{\mathbf{12}}\left|{\mathbf{0}}_{\mathbf{13}}\right|\dots \right]\ \left( \mod\ 7\right):\mbox{\fontencoding{T1}\fontfamily{cmr}\selectfont\char"0E} {\tilde{\mathrm{A}}}_{\mathrm{m}}\mbox{\fontencoding{T1}\fontfamily{cmr}\selectfont\char"0F} *\mbox{\fontencoding{T1}\fontfamily{cmr}\selectfont\char"0E} {\tilde{\mathrm{A}}}_{\mathrm{z}}\mbox{\fontencoding{T1}\fontfamily{cmr}\selectfont\char"0F} =\mbox{\fontencoding{T1}\fontfamily{cmr}\selectfont\char"0E} {\mathring{\mathrm{A}}}_{\mathrm{m}}\mbox{\fontencoding{T1}\fontfamily{cmr}\selectfont\char"0F} . $$

(see Appendix E for details)

$$ = \left[{2}_1\left|{5}_2\right|{3}_3\left|{0}_4\right|{3}_5\left|{6}_6\right|{2}_7\left|{0}_8\right|{1}_9\left|\left|{\mathbf{0}}_{\mathbf{10}}\right|{\mathbf{0}}_{\mathbf{11}}\right|{\mathbf{0}}_{\mathbf{12}}\Big|\dots \right]\ \left( \mod\ 7\right). $$

Examples including formulae (17) − (21) in the first row are given in Table 7.

**Table 7:** Presentation of examples of the severity assessment after the modification of Criterion A

Symptom number	1	2	3	4	5 (fear)	6	7	8	9		Episode
row 1	2	5	3	0	3	6	2	0	1	(mod 7)	1
row 2	0	2	0	0	0	4	0	0	3	(mod 7)	0
row 3	0	0	0	5	4	0	0	0	0	(mod 7)	0
row 4	5	4	0	2	3	1	0	3	6	(mod 7)	1
row 5	0	6	4	1	0	0	0	0	2	(mod 7)	0
row 6	1	5	3	0	1	1	4	0	1	(mod 7)	1
row 7 (= row 4)	5	4	0	2	3	1	0	3	6	(mod 7)	1
row 8 (= row 2)	0	2	0	0	0	4	0	0	3	(mod 7)	0
row 9	0	6	4	1	5	0	0	0	2	(mod 7)	1
row 10	5	4	0	2	6	1	0	3	6	(mod 7)	1
Total sum	18	38	14	13	25	18	6	9	30		6
Average	1.8	3.8	1.4	1.3	2.5	1.8	0.6	0.9	3.0		0.6

Examples of symptom severity after modification of Criterion A are presented. A new item ‘fear’ was incorporated between symptoms 4 and 5, and the ninth symptom ‘diminished ability’ (eighth symptom in the original Criterion A) was deleted. A compressed expression similar to that in Table 4 can be given: {2∙‹A›_540231036_(4,7), 2∙‹A›_020004003_(2,8), ‹A›_540261036_(10),…}. Note that the components of rows 2 and 8 are equivalent, as are those of rows 4 and 7. Similar to the case for Table 4, the number of rows does not exceed 7^×9^

Additionally, a short manipulation from ‹ A_j_ › → ‹ Å_m_ › (i.e., (7) → (21)) is simply obtained by ‹A_w_› operating on ‹A_j_›, where

22 $$ \mbox{\fontencoding{T1}\fontfamily{cmr}\selectfont\char"0E} {\mathrm{A}}_{\mathrm{w}}\mbox{\fontencoding{T1}\fontfamily{cmr}\selectfont\char"0F} =\left[{0}_1\left|{0}_2\right|{0}_3\left|{0}_4\right|{4}_5\left|{4}_6\right|{2}_7\left|{3}_8\right|{0}_9\left|\left|{\mathbf{0}}_{\mathbf{10}}\right|{\mathbf{0}}_{\mathbf{11}}\right|{\mathbf{0}}_{\mathbf{12}}\Big|\dots \right]: \mbox{\fontencoding{T1}\fontfamily{cmr}\selectfont\char"0E} {\mathrm{A}}_{\mathrm{j}}\mbox{\fontencoding{T1}\fontfamily{cmr}\selectfont\char"0F} *\mbox{\fontencoding{T1}\fontfamily{cmr}\selectfont\char"0E} {\mathrm{A}}_{\mathrm{w}}\mbox{\fontencoding{T1}\fontfamily{cmr}\selectfont\char"0F} =\mbox{\fontencoding{T1}\fontfamily{cmr}\selectfont\char"0E} {\mathring{A}}_{\mathrm{m}}\mbox{\fontencoding{T1}\fontfamily{cmr}\selectfont\char"0F} . $$

(See Appendix F for details.)

In this manner, all assessments of Criterion A for a ‘major depressive episode’ in DSM-5 can be expressed and treated employing modulo-2 and −7 operations and the incorporation of 0 with conversion between the implicit 0 and explicit 0, or the deletion of implicit 0 accompanied by operations.

### 5. Examples of rerating a seven-point score via a non-paired assessment of the upgraded criterion and other independent assessments

We now turn to the upgrading of criteria or scales for depression as another aspect of Criterion A based on modulo-2 arithmetic Z_2_^×9^.

We consider the following scenario. The results of a certain assessment are expressed as ‹A_j_› (7) and are conjunctive with the criterion/indication for symptom selection of A_j_ (6). The operation of the indication for selection of ‘which items should be effective’ on the identity indication A_0_ (= [0_1_|0_2_|0_3_|0_4_|0_5_|0_6_|0_7_|0_8_|0_9_||**0**_**10**_|**0**_**11**_|**0**_**12**_|…] (mod 2); to select nothing for symptoms as being effective) (8) is upgraded from A_j_ (= A_0→j_) to A_r_ (= A_0→r_) (an upgraded scale). In other words, an indication A_j_ (6) for the selection of an item as being effective could be the result of an operation on A_0_:

$$ {\mathrm{A}}_0*\ {\mathrm{A}}_{\mathrm{j}}=\left[{0}_1\left|{0}_2\right|{0}_3\left|{0}_4\right|{0}_5\left|{0}_6\right|{0}_7\left|{0}_8\right|{0}_9\left|\left|{\mathbf{0}}_{\mathbf{10}}\right|{\mathbf{0}}_{\mathbf{11}}\right|{\mathbf{0}}_{\mathbf{12}}\Big|\dots \right] + \left[{1}_1\left|{1}_2\right|{1}_3\left|{0}_4\right|{1}_5\left|{1}_6\right|{0}_7\left|{1}_8\right|{1}_9\left|\left|{\mathbf{0}}_{\mathbf{10}}\right|{\mathbf{0}}_{\mathbf{11}}\right|{\mathbf{0}}_{\mathbf{12}}\Big|\dots \right]\ \left( \mod\ 2\right) = \left[{1}_1\left|{1}_2\right|{1}_3\left|{0}_4\right|{1}_5\left|{1}_6\right|{0}_7\left|{1}_8\right|{1}_9\left|\left|{\mathbf{0}}_{\mathbf{10}}\right|{\mathbf{0}}_{\mathbf{11}}\right|{\mathbf{0}}_{\mathbf{12}}\Big|\dots \right]\ \left( \mod\ 2\right) = {\mathrm{A}}_{\mathrm{j}}.\kern15em (6) $$

(The first, second, third, fifth, sixth, eight and ninth items are effective on the scale A_j_; the operation is to select these items as being effective on Criterion A.)

Naturally, an operation A_k_ that changes the indication for A_j_ can be considered. Let A_k_ = [1_1_|1_2_|0_3_|1_4_|0_5_|1_6_|1_7_|0_8_|1_9_||**0**_**10**_|**0**_**11**_|**0**_**12**_|…]. The indication for item selection as being effective A_j_ * A_k_ (= A_l_) is calculated as

23 $$ {\mathrm{A}}_{\mathrm{j}}*\ {\mathrm{A}}_{\mathrm{k}}=\left[{1}_1\left|{1}_2\right|{1}_3\left|{0}_4\right|{1}_5\left|{1}_6\right|{0}_7\left|{1}_8\right|{1}_9\left|\left|{\mathbf{0}}_{\mathbf{10}}\right|{\mathbf{0}}_{\mathbf{11}}\right|{\mathbf{0}}_{\mathbf{12}}\Big|\dots \right] + \left[{1}_1\left|{1}_2\right|{0}_3\left|{1}_4\right|{0}_5\left|{1}_6\right|{1}_7\left|{0}_8\right|{1}_9\left|\left|{\mathbf{0}}_{\mathbf{10}}\right|{\mathbf{0}}_{\mathbf{11}}\right|{\mathbf{0}}_{\mathbf{12}}\Big|\dots \right]\ \left( \mod\ 2\right) = \left[{0}_1\left|{0}_2\right|{1}_3\left|{1}_4\right|{1}_5\left|{0}_6\right|{1}_7\left|{1}_8\right|{0}_9\left|\left|{\mathbf{0}}_{\mathbf{10}}\right|{\mathbf{0}}_{\mathbf{11}}\right|{\mathbf{0}}_{\mathbf{12}}\Big|\dots \right]\ \left( \mod\ 2\right) = {\mathrm{A}}_{\mathrm{l}}\left( \mod\ 2\right). $$

(The contents of indication A_l_ are that the third, fourth, fifth, seventh and eight items are effective on the scale A_l_; the operation is to select these items as being effective on Criterion A.)

As seen above, the indication for item selection A_j_ has dual meanings: 1) to select specific items rated by a value of 1 as effective symptoms and 2) to change the indication between 0 and 1.

We then interpret the operator A_j_ as A_j_ * A_0_ and the operator A_r_ as A_r_ * A_0_. The score of ‹A_j_› is transformed to ‹A_r_› via ‹A_j_› ∙ (A_r_ * A_0_) (mod 7); i.e.,

for arbitrary j and r,

24 $$ \mbox{\fontencoding{T1}\fontfamily{cmr}\selectfont\char"0E} {\mathrm{A}}_{\mathrm{r}} \mbox{\fontencoding{T1}\fontfamily{cmr}\selectfont\char"0F} \left( \mod\ 7\right) = {\mathrm{A}}_{\mathrm{r}}\cdot \mbox{\fontencoding{T1}\fontfamily{cmr}\selectfont\char"0E} {\mathrm{A}}_{\mathrm{j}}\mbox{\fontencoding{T1}\fontfamily{cmr}\selectfont\char"0F} \left( \mod\ 7\right) = \mbox{\fontencoding{T1}\fontfamily{cmr}\selectfont\char"0E} {\mathrm{A}}_{\mathrm{j}}\mbox{\fontencoding{T1}\fontfamily{cmr}\selectfont\char"0F} \cdot {\mathrm{A}}_{\mathrm{r}}\left( \mod\ 7\right). $$

Expression (24) is similar to (5) and can be used to present the scored result on a certain subscale employing only multiplication between the indication vector for item determination and the seven-point score for patient evaluation under the condition that the index numbers are trimmed correctly. Manipulations such as (17) − (22) via the incorporation/deletion of 0 s are necessary to match the index numbers. Here, it is not assured that 0 s are paired between A_j_ and ‹A_j_›.

We consider a situation that the criterion is upgraded from A_j_ to A_r_ by selecting symptoms

25 $$ {\mathrm{A}}_{\mathrm{r}}=\left[{\sigma}_1\left|{\sigma}_2\right|{\sigma}_3\left|{\sigma}_4\right|{\sigma}_5\left|{\sigma}_6\right|{\sigma}_7\left|{\sigma}_8\right|{\sigma}_9\left|\left|{\mathbf{0}}_{\mathbf{10}}\right|{\mathbf{0}}_{\mathbf{11}}\right|{\mathbf{0}}_{\mathbf{12}}\Big|\dots \right]\ \left( \mod\ 2\right),\ \mathrm{where}\ {\sigma}_{\mathrm{i}} = \left\{0\ \mathrm{or}\ 1\right\}, $$

And

26 $$ \mbox{\fontencoding{T1}\fontfamily{cmr}\selectfont\char"0E} {\mathrm{A}}_{\mathrm{j}}\mbox{\fontencoding{T1}\fontfamily{cmr}\selectfont\char"0F} =\left[\mbox{\fontencoding{T1}\fontfamily{cmr}\selectfont\char"0E} {\mathrm{a}}_{\left(\mathrm{j}\right)1}\mbox{\fontencoding{T1}\fontfamily{cmr}\selectfont\char"0F} \left|\mbox{\fontencoding{T1}\fontfamily{cmr}\selectfont\char"0E} {\mathrm{a}}_{\left(\mathrm{j}\right)2}\mbox{\fontencoding{T1}\fontfamily{cmr}\selectfont\char"0F} \right|\mbox{\fontencoding{T1}\fontfamily{cmr}\selectfont\char"0E} {\mathrm{a}}_{\left(\mathrm{j}\right)3}\mbox{\fontencoding{T1}\fontfamily{cmr}\selectfont\char"0F} \left|\mbox{\fontencoding{T1}\fontfamily{cmr}\selectfont\char"0E} {\mathrm{a}}_{\left(\mathrm{j}\right)4}\mbox{\fontencoding{T1}\fontfamily{cmr}\selectfont\char"0F} \right|\mbox{\fontencoding{T1}\fontfamily{cmr}\selectfont\char"0E} {\mathrm{a}}_{\left(\mathrm{j}\right)5}\mbox{\fontencoding{T1}\fontfamily{cmr}\selectfont\char"0F} \left|\mbox{\fontencoding{T1}\fontfamily{cmr}\selectfont\char"0E} {\mathrm{a}}_{\left(\mathrm{j}\right)6}\mbox{\fontencoding{T1}\fontfamily{cmr}\selectfont\char"0F} \right|\mbox{\fontencoding{T1}\fontfamily{cmr}\selectfont\char"0E} {\mathrm{a}}_{\left(\mathrm{j}\right)7}\mbox{\fontencoding{T1}\fontfamily{cmr}\selectfont\char"0F} \left|\mbox{\fontencoding{T1}\fontfamily{cmr}\selectfont\char"0E} {\mathrm{a}}_{\left(\mathrm{j}\right)8}\mbox{\fontencoding{T1}\fontfamily{cmr}\selectfont\char"0F} \right|\mbox{\fontencoding{T1}\fontfamily{cmr}\selectfont\char"0E} {\mathrm{a}}_{\left(\mathrm{j}\right)9}\mbox{\fontencoding{T1}\fontfamily{cmr}\selectfont\char"0F} \left|\left|{\mathbf{0}}_{\mathbf{10}}\right|{\mathbf{0}}_{\mathbf{11}}\right|{\mathbf{0}}_{\mathbf{12}}\Big|\dots \right]\ \left( \mod\ 7\right), $$

which are obtained independently.

Hence, according to (24),

27 $$ \mbox{\fontencoding{T1}\fontfamily{cmr}\selectfont\char"0E} {\mathrm{A}}_{\mathrm{r}}\mbox{\fontencoding{T1}\fontfamily{cmr}\selectfont\char"0F} =\mbox{\fontencoding{T1}\fontfamily{cmr}\selectfont\char"0E} {\mathrm{A}}_{\mathrm{j}}\mbox{\fontencoding{T1}\fontfamily{cmr}\selectfont\char"0F} \cdot {\mathrm{A}}_{\mathrm{r}}=\left[\mbox{\fontencoding{T1}\fontfamily{cmr}\selectfont\char"0E} {\mathrm{a}}_{\left(\mathrm{j}\right)1}\mbox{\fontencoding{T1}\fontfamily{cmr}\selectfont\char"0F} \cdot {\sigma}_1\left|\mbox{\fontencoding{T1}\fontfamily{cmr}\selectfont\char"0E} {\mathrm{a}}_{\left(\mathrm{j}\right)2}\mbox{\fontencoding{T1}\fontfamily{cmr}\selectfont\char"0F} \cdot {\sigma}_2\right|\mbox{\fontencoding{T1}\fontfamily{cmr}\selectfont\char"0E} {\mathrm{a}}_{\left(\mathrm{j}\right)3}\mbox{\fontencoding{T1}\fontfamily{cmr}\selectfont\char"0F} \cdot {\sigma}_3\left|\mbox{\fontencoding{T1}\fontfamily{cmr}\selectfont\char"0E} {\mathrm{a}}_{\left(\mathrm{j}\right)4}\mbox{\fontencoding{T1}\fontfamily{cmr}\selectfont\char"0F} \cdot {\sigma}_4\right|\mbox{\fontencoding{T1}\fontfamily{cmr}\selectfont\char"0E} {\mathrm{a}}_{\left(\mathrm{j}\right)5}\mbox{\fontencoding{T1}\fontfamily{cmr}\selectfont\char"0F} \cdot {\sigma}_5\left|\mbox{\fontencoding{T1}\fontfamily{cmr}\selectfont\char"0E} {\mathrm{a}}_{\left(\mathrm{j}\right)6}\mbox{\fontencoding{T1}\fontfamily{cmr}\selectfont\char"0F} \cdot {\sigma}_6\right|\mbox{\fontencoding{T1}\fontfamily{cmr}\selectfont\char"0E} {\mathrm{a}}_{\left(\mathrm{j}\right)7}\mbox{\fontencoding{T1}\fontfamily{cmr}\selectfont\char"0F} \cdot {\sigma}_7\left|\mbox{\fontencoding{T1}\fontfamily{cmr}\selectfont\char"0E} {\mathrm{a}}_{\left(\mathrm{j}\right)8}\mbox{\fontencoding{T1}\fontfamily{cmr}\selectfont\char"0F} \cdot {\sigma}_8\right|\mbox{\fontencoding{T1}\fontfamily{cmr}\selectfont\char"0E} {\mathrm{a}}_{\left(\mathrm{j}\right)9}\mbox{\fontencoding{T1}\fontfamily{cmr}\selectfont\char"0F} \cdot {\sigma}_9\left|\left|{\mathbf{0}}_{\mathbf{10}}\right|{\mathbf{0}}_{\mathbf{11}}\right|{\mathbf{0}}_{\mathbf{12}}\Big|\dots \right]\ \left( \mod\ 7\right) $$

(a state of severity in the r-th session on Criterion A for a certain patient, determined by the indication for item selection (25)).

28 $$ \mathrm{Suppose}\ {\mathrm{A}}_{\mathrm{r}}=\left[{1}_1\left|{0}_2\right|{1}_3\left|{0}_4\right|{1}_5\left|{1}_6\right|{1}_7\left|{0}_8\right|{1}_9\left|\left|{\mathbf{0}}_{\mathbf{10}}\right|{\mathbf{0}}_{\mathbf{11}}\right|{\mathbf{0}}_{\mathbf{12}}\Big|\dots \right]\ \left( \mod\ 2\right); $$

i.e., the first, third, fifth, sixth, seventh and ninth items are effective on the scale A_r_; i.e., σ_i_ = 1 (for i = 1, 3, 5, 6, 7, 9) and σ_i_ = 0 (for i = 2, 4, 8, 10, 11,…) in (25) and (27). At the same time, let ‹A_j_› be such that

29 $$ \mbox{\fontencoding{T1}\fontfamily{cmr}\selectfont\char"0E} {\mathrm{A}}_{\mathrm{j}}\mbox{\fontencoding{T1}\fontfamily{cmr}\selectfont\char"0F} =\left[{2}_1\left|{5}_2\right|{3}_3\left|{0}_4\right|{6}_5\left|{2}_6\right|{0}_7\left|{4}_8\right|{1}_9\left|\left|{\mathbf{0}}_{\mathbf{10}}\right|{\mathbf{0}}_{\mathbf{11}}\right|{\mathbf{0}}_{\mathbf{12}}\Big|\dots \right]\ \left( \mod\ 7\right). $$

From (24) and (27) − (29), it follows that

30 $$ \mbox{\fontencoding{T1}\fontfamily{cmr}\selectfont\char"0E} {\mathrm{A}}_{\mathrm{r}}\mbox{\fontencoding{T1}\fontfamily{cmr}\selectfont\char"0F} =\mbox{\fontencoding{T1}\fontfamily{cmr}\selectfont\char"0E} {\mathrm{A}}_{\mathrm{j}}\mbox{\fontencoding{T1}\fontfamily{cmr}\selectfont\char"0F} \cdot {\mathrm{A}}_{\mathrm{r}}\left( \mod\ 7\right)=\left[{2}_1\left|{5}_2\right|{3}_3\left|{0}_4\right|{6}_5\left|{2}_6\right|{0}_7\left|{4}_8\right|{1}_9\left|\left|{\mathbf{0}}_{\mathbf{10}}\right|{\mathbf{0}}_{\mathbf{11}}\right|{\mathbf{0}}_{\mathbf{12}}\Big|\dots \right]\cdot \left[{1}_1\left|{0}_2\right|{1}_3\left|{0}_4\right|{1}_5\left|{1}_6\right|{1}_7\left|{0}_8\right|{1}_9\left|\left|{\mathbf{0}}_{\mathbf{10}}\right|{\mathbf{0}}_{\mathbf{11}}\right|{\mathbf{0}}_{\mathbf{12}}\Big|\dots \right]\left( \mod\ 7\right)=\left[{2}_1\left|{0}_2\right|{3}_3\left|{0}_4\right|{6}_5\left|{2}_6\right|{0}_7\left|{4}_8\right|{1}_9\left|\left|{\mathbf{0}}_{\mathbf{10}}\right|{\mathbf{0}}_{\mathbf{11}}\right|{\mathbf{0}}_{\mathbf{12}}\Big|\dots \right]\ \left( \mod\ 7\right). $$

(= [21|5_2_|33|0_4_|65|26|07|4_8_|19||**0**_**10**_|**0**_**11**_|**0**_**12**_|…] (mod 7) where s are effective)

Examples are given in Table 8, including formula (30) in the first row. Here, an optional diagnosis is definable, provided that appropriate rules are given (see the legend of Table 8).

**Table 8:** Presentation of examples of further modified severity assessment on Criterion A

Symptom number	1	2	3	4	5	6	7	8	9		Modified episode
row 1 (= {‹A_1_›∙A_r_})	2	5	3	0	6	2	0	4	1	(mod 7)	1
row 2 (={‹A_2_›∙A_r_})	0	2	0	0	4	0	0	2	3	(mod 7)	0
row 3 (= {‹A_3_›∙A_r_})	0	0	0	5	0	0	0	3	0	(mod 7)	0
row 4 (= {‹A_4_›∙A_r_})	5	4	0	2	1	0	3	0	6	(mod 7)	0
row 5 (= {‹A_5_›∙A_r_})	0	6	4	1	0	0	0	0	2	(mod 7)	0
row 6 (= {‹A_6_›∙A_r_})	1	5	3	0	1	4	0	0	1	(mod 7)	1
row 7 (= row 4) (= {‹A_7_›∙A_r_})	5	4	0	2	1	0	3	0	6	(mod 7)	0
row 8 (= row 2) (= {‹A_8_›∙A_r_})	0	2	0	0	4	0	0	2	3	(mod 7)	0
row 9 (= {‹A_9_›∙A_r_})	0	6	4	1	0	0	0	0	2	(mod 7)	0
row 10 (= row 4) (= {‹A_10_›∙A_r_})	5	4	0	2	1	0	3	0	6	(mod 7)	0
Total sum	18	38	14	13	18	6	9	11	30		5
Averages	1.8	3.8	1.4	1.3	1.8	0.6	0.9	1.1	3.0		0.2

The indication for item selection as being effective; A_r_ = [1_1_|0_2_|1_3_|0_4_|1_5_|1_6_|1_7_|0_8_|1_9_||**0**_**10**_|**0**_**11**_|**0**_**12**_|…] (mod 2) over Table 3

A further modified (sub)scale based on Criterion A is presented. Here, the first, third, sixth, seventh and ninth items are effective. The order of effectiveness via selection of items is A_r_ = [σ_1_|σ_2_|σ_3_|σ_4_|σ_5_|σ_6_|σ_7_|σ_8_|σ_9_||**0**_**10**_|**0**_**11**_|**0**_**12**_|…] (mod 2) = [1_1_|0_2_|1_3_|0_4_|1_5_|1_6_|1_7_|0_8_|1_9_||**0**_**10**_|**0**_**11**_|**0**_**12**_|…] (mod 2). If the j-th row (in this case, j = 1,2,3,…10) in Table 3 is expressed as ‹A_j_›, then the modified (sub)scale is provided by the inner product ‹A_j_› ∙ A_r_, where effective items are denoted by the value 1 and ineffective items by the value 0. The omitted display for the j-th row is given by {‹A_j_› ∙ A_r_}. If the components with σ_i_ = 0 are used at this time; however, these ineffective results of assessments are expected to be stored implicitly and can be pulled out as explicit data when needed in, for example, a future upgrade of criteria of the DSM. The extreme right column indicates the presence/absence of a ‘modified episode’ relating to item selection of Criterion A when the episode needs four of six symptoms for the diagnosis, with at least one of them being symptom (1)

The meaning of expression (30) is that, according to the upgrading (modification) of criterion A_j_ → A_r_, the score on scale ‹A_j_› is transformed to that on ‹A_r_›.

Herein, we use the following notation to describe various cases. We denote by {A_j_} a Criterion A_j_ where unselected items are given a value 0 at A_r_ (e.g., (28)), except the trailing series of 0 s that are implicitly indicated while the place number indexing is retained; i.e.,

31 $$ \left\{{\mathrm{A}}_{\mathrm{r}}\right\} = \left[{1}_1\left|{1}_3\right|{1}_5\left|{1}_6\right|{1}_7\left|{1}_9\right|\left|{\mathbf{0}}_{10}\right|{\mathbf{0}}_{11}\left|{\mathbf{0}}_{12}\right|\dots \right]\ \left( \mod\ 2\right). $$

Therefore, σ_i_ = 1 (for i = 1, 3, 5, 6, 7, 9) and σ_i_ = 0 (for i = 2, 4, 8, 10, 11,…) in (25) and (27). Here, the explicitly indicated place numbers (for i = 1, 3, 5, 6, 7, 9) are the same as in (28) and missing subscripted place numbers indicate omitted 0 s. Hence, (31) without trailing 0 s and subscripts represents an ordinal/finite Criterion A_r_. (Note that although the omitted items are not used at this time, they could be pulled out freely when needed in, for example, a future upgrade of the DSM.)

Likewise, following from (27) and (28), for ‹A_r_›, a similar omitted display for the results of assessment due to A_r_ is obtained as

32 $$ \left\{\mbox{\fontencoding{T1}\fontfamily{cmr}\selectfont\char"0E} {\mathrm{A}}_{\mathrm{r}}\mbox{\fontencoding{T1}\fontfamily{cmr}\selectfont\char"0F} \right\}=\left\{\mbox{\fontencoding{T1}\fontfamily{cmr}\selectfont\char"0E} {\mathrm{A}}_{\mathrm{j}}\mbox{\fontencoding{T1}\fontfamily{cmr}\selectfont\char"0F} \cdot {\mathrm{A}}_{\mathrm{r}}\ \left(\kern-0.25em , \mod\ 7\right)\right\}=\left[\mbox{\fontencoding{T1}\fontfamily{cmr}\selectfont\char"0E} {\mathrm{a}}_{\left(\mathrm{r}\right)1}\mbox{\fontencoding{T1}\fontfamily{cmr}\selectfont\char"0F} \cdot 1\left|\mbox{\fontencoding{T1}\fontfamily{cmr}\selectfont\char"0E} {\mathrm{a}}_{\left(\mathrm{r}\right)2}\mbox{\fontencoding{T1}\fontfamily{cmr}\selectfont\char"0F} \cdot 0\right|\mbox{\fontencoding{T1}\fontfamily{cmr}\selectfont\char"0E} {\mathrm{a}}_{\left(\mathrm{r}\right)3}\mbox{\fontencoding{T1}\fontfamily{cmr}\selectfont\char"0F} \cdot 1\left|\mbox{\fontencoding{T1}\fontfamily{cmr}\selectfont\char"0E} {\mathrm{a}}_{\left(\mathrm{r}\right)4}\mbox{\fontencoding{T1}\fontfamily{cmr}\selectfont\char"0F} \cdot 0\right|\mbox{\fontencoding{T1}\fontfamily{cmr}\selectfont\char"0E} {\mathrm{a}}_{\left(\mathrm{r}\right)5}\mbox{\fontencoding{T1}\fontfamily{cmr}\selectfont\char"0F} \cdot 1\left|\mbox{\fontencoding{T1}\fontfamily{cmr}\selectfont\char"0E} {\mathrm{a}}_{\left(\mathrm{r}\right)6}\mbox{\fontencoding{T1}\fontfamily{cmr}\selectfont\char"0F} \cdot 1\right|\mbox{\fontencoding{T1}\fontfamily{cmr}\selectfont\char"0E} {\mathrm{a}}_{\left(\mathrm{r}\right)7}\mbox{\fontencoding{T1}\fontfamily{cmr}\selectfont\char"0F} \cdot 1\left|\mbox{\fontencoding{T1}\fontfamily{cmr}\selectfont\char"0E} {\mathrm{a}}_{\left(\mathrm{r}\right)8}\mbox{\fontencoding{T1}\fontfamily{cmr}\selectfont\char"0F} \cdot 0\right|\mbox{\fontencoding{T1}\fontfamily{cmr}\selectfont\char"0E} {\mathrm{a}}_{\left(\mathrm{r}\right)9}\mbox{\fontencoding{T1}\fontfamily{cmr}\selectfont\char"0F} \cdot 1\left|\right|{\mathbf{0}}_{\mathbf{10}}\left|{\mathbf{0}}_{\mathbf{11}}\right|{\mathbf{0}}_{\mathbf{12}}\Big|\dots \right]\kern0.5em \left(\kern-0.25em , \mod\ 7\right)=\left[\mbox{\fontencoding{T1}\fontfamily{cmr}\selectfont\char"0E} {\mathrm{a}}_{\left(\mathrm{r}\right)1}\mbox{\fontencoding{T1}\fontfamily{cmr}\selectfont\char"0F} \left|\mbox{\fontencoding{T1}\fontfamily{cmr}\selectfont\char"0E} {\mathrm{a}}_{\left(\mathrm{r}\right)3}\mbox{\fontencoding{T1}\fontfamily{cmr}\selectfont\char"0F} \right|\mbox{\fontencoding{T1}\fontfamily{cmr}\selectfont\char"0E} {\mathrm{a}}_{\left(\mathrm{r}\right)5}\mbox{\fontencoding{T1}\fontfamily{cmr}\selectfont\char"0F} \left|\mbox{\fontencoding{T1}\fontfamily{cmr}\selectfont\char"0E} {\mathrm{a}}_{\left(\mathrm{r}\right)6}\mbox{\fontencoding{T1}\fontfamily{cmr}\selectfont\char"0F} \right|\mbox{\fontencoding{T1}\fontfamily{cmr}\selectfont\char"0E} {\mathrm{a}}_{\left(\mathrm{r}\right)7}\mbox{\fontencoding{T1}\fontfamily{cmr}\selectfont\char"0F} \left|\mbox{\fontencoding{T1}\fontfamily{cmr}\selectfont\char"0E} {\mathrm{a}}_{\left(\mathrm{r}\right)9}\mbox{\fontencoding{T1}\fontfamily{cmr}\selectfont\char"0F} \right|\left|{\mathbf{0}}_{\mathbf{10}}\right|{\mathbf{0}}_{\mathbf{11}}{\mathbf{0}}_{\mathbf{12}}\Big|\dots \right]\kern0.5em \left( \mod\ 7\right) $$

33 $$ =\left[{2}_1\left|{3}_3\right|{3}_5\left|{6}_6\right|{2}_7\left|{1}_9\right|\left|{\mathbf{0}}_{\mathbf{10}}\right|{\mathbf{0}}_{\mathbf{11}}\left|{\mathbf{0}}_{\mathbf{12}}\right|\dots \right]\ \left( \mod\ 7\right), $$

where ‹a_(r)i_› = {0, 1, 2, 3, 4, 5, 6} (r: number of sessions; *r* = 1, 2, 3,…), (i: number of components; *i* = 1, 2, 3,…). Examples are given in Table 8, including the parts of formula (33) colored in blue in the first row. Expressions (32) and (33) express the results of rating at a subscale selected by A_r_ according to (28) and (31).

Optionally, using modulo arithmetic (especially ‘addition’), it is considered possible to rate the degree of unstandardized assessment of a certain psychiatrist or to highlight specific scores in either an overview or focused investigation of the data. One such attempt is demonstrated in Table 9.

**Table 9:** Illustration highlighting incongruent scores on Criterion A

Symptom number	1	2	3	4	5	6	7	8	9		Episode
row 1	0	0	1	1	0	1	1	1	0	(mod 2)	0
row 2	1	0	0	1	0	0	1	1	0	(mod 2)	1
row 3	1	1	0	0	1	0	1	1	1	(mod 2)	1
row 4	1	1	0	1	1	0	1	0	1	(mod 2)	1
row 5	1	0	1	0	1	0	1	0	0	(mod 2)	1
row 6	0	0	1	1	0	1	1	0	0	(mod 2)	0
row 7	0	0	0	0	0	0	0	0	0	(mod 2)	0
row 8	1	0	0	1	0	0	1	1	0	(mod 2)	1
row 9	1	0	1	0	1	0	1	0	0	(mod 2)	1
row 10	0	0	0	0	0	0	0	0	0	(mod 2)	0
Total sum (except for row 4)	5	1	4	4	3	2	7	4	1		5
Average (except for row 4)	0.5	0.1	0.4	0.4	0.3	0.2	0.7	0.4	0.1		0.5

By specifying/focusing on an arbitral j-th row (j = 1, 2, 3,…), and by adding the components of the j-th row to all other rows individually, the components for which scores (meaning presence/absence of symptoms, effectiveness/ineffectiveness of symptoms and so on) are different from those of the j-th row can be highlighted by values of 1 (and the components equivalent to those of the j-th row can be denoted 0) according to modulo-2 arithmetic (especially, addition). If a total of 10 diagnoses are made by 10 psychiatrists for the same patient in the same session, the count of values of 1 indicates the degree of fluctuation of standardization of the j-th assessment (j-th psychiatrist). Row (patient/session) 4 in Table 1 is taken as an example (highlighted in silver). Apart from the extreme right column, 31 (count of the value 1) divided by 81 cells (= ‘10 – 1’ (rows) × 9 (items)) (that for the other nine psychiatrists; except for 4-th row) = 31/81 = 0.3827 = 38.27 (%) could be regarded as a ratio for unstandardization of the j-th assessment (psychiatrist). In the extreme right column, a value of 0 (or 1) means the equivalency (non-equivalency) of the respective diagnosis for an episode to the highlighted case (in this case, row 4); this also obeys modulo-2 addition

In summary, the original Criterion A (denoted A_j_) for a ‘major depressive episode’ in DSM-5 can be used as a simple diagnosis under modulo-2 operation. At the same time, the modified Criterion A (denoted A_r_) can be used to display a modified score for severity of a patient’s state (denoted ‹A_r_›). We believe that A_j_ and A_r_ are also applicable as an order that asks psychiatrists ‘to rate specific symptoms’, such as ‘to assess the ‘i, j, k-th’ items on the modified scale of ‹A_r_›’, where a similar vector could be defined. Furthermore, various themes (‘record’, ‘cover’, ‘highlight’, ‘i, j, k-th’ items) are definable so long as they have binominal meanings such as {0, 1}. The combined use of an M-tuple is possible depending on further devises; i.e., A_j_^×M^ = [Z_2_^×M^_1_| Z_2_^×M^_2_| Z_2_^×M^_3_|…Z_2_^×M^_i_|…] (mod 2) (M: number of optional rules). For instance, if there are three (M = 3) optional assessments such as ‘select/delete’, ‘record’, and ‘cover’, then A_j_^×3^ = [Z_2_^×3^_1_| Z_2_^×3^_2_| Z_2_^×3^_3_|…Z_2_^×3^_i_|…] (mod 2) = [(1,0,1)_1_| (0,1,1)_2_| (1,1,1)_3_|…|(1,1,0)_i_|…|**(0,0,0)**_**N+1**_|**(0,0,0)**_**N+2**_|**(0,0,0)**_**N+3**_|…] (mod 2) can express the combined order for the practice for psychiatrists although practical application is unclear at this stage.

## Discussion

The present study found that the simple structure of the DSM-5 system can be systemized in a more rational form that is close to a mathematical formalism. Binominal characteristics allow simple composition under modulo-2 operation (§1). All vectors can be included in a single set using explicit 0 s and implicit 0 s that are denoted with and without index numbers respectively (§2). Vectors have dual meanings similar to those of a positional vector. A value of 1 for a component i (a_(j)i_ = {0 or 1}) expresses the presence/conversion of a certain symptom whereas a value of 0 expresses absence/non-conversion (§3). A seven-point scoring appears optimal (§4). Each value (e.g., 3) of a component can then express the severity of a symptom. Essentially, we adopt this interpretation without advice. However, a value could also express the degree of change in the symptoms of Criterion A via item selection (e.g., 0 → 1), or the severity, from an identity state via a rating (e.g., 0 → 3) (§4, §5). Then, optionally, the emergence of a certain symptom for the i-th component is expressed as 1_i_ and realized by 0_i_ → 1_i_. The action of 1_i_ on 0_i_ then means the deletion of the symptom 1_i_ → 0_i_ because 1 + 1 = 0 (mod 2). In other words, the former example expresses the ‘absolutely present’ state while the latter expresses the ‘indication for conversion’ between presence and absence; i.e., 0_i_↔1_i_. A value 0_i_ for component i can mean there is no conversion between 0 and 1 at the i-th item in the upgraded Criterion A (§4, §5). The values 0_i_ and 1_i_ could also indicate conversion between a selected/unselected i-th item on the upgraded criterion/scale. In this way, the dual meaning of the vector (positional vector) allows application of cyclic symmetry to any diagnosis and upgrading criterion/scale in DSM-5.

The difference between an explicit 0 and implicit 0 is whether the 0 is written as a numeral followed by an index. In fact, a non-zero number can be incorporated into any interval in (6) or (7), and there are operators that can induce changes only within modulo addition. For example, in the latter case, we can incorporate an item with the value 5 between the fourth and fifth items of ‹A_j_›. However, this enforced use inevitably results in a non-ignorable problem in interpretation for manipulation because a state of patients in which there is a sudden emergence of non-zero severity (e.g., […|2|5|1|…]) cannot be regarded as being based on the same state of patients as before the change (e.g., […|2||1|…]). These two states with/without a sudden emergence of non-zero intensity (in the above case; 5) relate to the revelation of a new symptom, which should be discriminated from the absence of the new symptom. In other words, the introduction of the optional concept (§2) for conversion from ‘implicit’ to ‘explicit’ is considered permissible only via a value of 0 because only a value of 0 can indicate ‘absence’ or ‘no change in the order of selecting symptoms’; i.e., only a value of 0 allows the recognized assessment to keep the same meaning. We thus permit the incorporation/deletion of items only via the value 0, which plays the role of a window for revealing or hiding an item. However, while implicit 0 means there is no assessment of a specific symptom, an explicit 0 means there is non-existence of the specific symptom. Importantly, in the psychiatric assessment of patients, the absence of a certain symptom is not equivalent to the disregard of its assessment. For instance, an obsessive symptom with ‘unreasonable thought’ has affinity with obsessive–compulsive disorder, while that without ‘unreasonable thought’ implies other atypical disorders [20], for which the drug treatment often differs. In this model, subjectively greater meaning might be assigned to symptoms that are recognized. This could introduce bias into the comprehension of the disease states. A difference in interpretation of explicit and implicit 0 might have such outcomes. As another example, in a clinical examination looking for infection with a certain influenza, a negative result (non-positive result) could be scored as 0, which would have positive meaning for the possible non-infection of influenza. However, if a score of 0 expresses deletion of that item, or an indication of no change, an item having a constant score of 0 might have little meaning. In both cases, the results do not affect evaluation/recognition from the standpoint that only non-zero symptoms or severities have positive meaning for the raters of the disease states. The interpretation of the explicit/implicit 0 is thus ambiguous to some extent; however, the roles played by the value of 0 cannot be played by other values such as 1, 2,…, 6. Therefore, this dual use of the value of 0 is necessary for the composition of a single set A that all vectors belong to. The other important issue is considered to be exhaustiveness, where no results obtained with the model deviate from existing theorems of abstract algebra. Briefly, when we consider an arbitral order A_k_ on A_j_, 1) the changes in item selection or effectiveness for severity are obtained by (A_j_ * A_k_) (mod 2) = (A_j_ + A_k_) (mod 2), 2) the modified criterion/diagnosis is calculated by (A_j_ ∙ A_k_) (mod 2) (‘∙’: inner product), and 3) the assessment for severity on a modified (sub)scale is provided as ‹A_j_› ∙ A_r_ (mod 7). Note that any diagnostic states containing symptom-assessment parts and operator parts could be directly linked (changed) within at least one operation owing to characteristics of the group/field/field for the code of the respective number (e.g., 2, 7) of modulo arithmetic (where a prime number is preferred) as was presented in our previous reports [9, 10, 14]. We believe that ideally data for symptom severity obtained via a completely linearized (calibrated) evaluation scale or criteria could be metric data. Unfortunately, our model does not contain methods for linearization of those scales or criteria. However, we believe that, in general, treatment of metric data such as those of “the Numerical rating scale (NRS; 11-point numerical pain rating scale)” [21, 22] is possible via modulo arithmetic because our model itself is not incompatible with the style of calibration itself so long as the ordinal/metric scales are accompanied by ‘0’ with no absolute need for a quantitative calibration that is reported in our previous study [14]. It is considered necessary that descriptions that are more compressed should be given or less data mining should be performed, especially in the medical field for diagnosis, prescribing, recording and data storing. We also believe that, with further improvement, the simple tool presented in the present article can serve in the recording or storing of clinical findings and results. We expect that a possible advantage of the proposed method is the reduced weighting or mining of clinical data.

The limitations of the proposed method should be noted. As is seen in the DSMs (especially DSMs III to 5), the number of diseases that can be diagnosed is increasing. Because Criterion A treated so far is only a part of DSM-5, our results only apply to the concept of the DSM system. The use of vector-like notations could be convenient for the overview of the state and characteristics; however, the notations do not ensure preciseness for diagnostic development. A larger number of items results in more similarity, more repetition, more confusion between items, and less internal reliability. This is because global states of disease cannot always be composed as the combination of components in principle.

Additionally, the importance of the present model to biological applications such as the use of biomarkers is not considered. In fact, the DSM has been revised by assimilating updated knowledge of science. The absorption and mining of important findings in medicine by integrating biology, psychosocial, pharmaceutical, and genomic methods into the proposed model are expected.

There is no clear method that collaborates with statistical methods, presently. However, the development of criteria and scales of practical disease states is considered helpful not only to clinical treatment but also to the systematization of clinical medicine, such that the level of formalism of clinical medicine approaches that of other fields of natural science such as chemistry, physics and mathematics.

## Conclusions

The symmetrical treatment of the diagnosis, rescoring and rescaling of depression according to DSM-5 criteria, although difficult, is considered possible using modulo arithmetic, especially modulo addition. Such treatment will allow the formalism level of clinical medicine to approach that of other fields of natural science.

## Appendix A

$$ \begin{array}{l}{\hat{\mathrm{A}}}_{\mathrm{k}}*\ {\hat{\mathrm{A}}}_{\mathrm{y}} = {\hat{\mathrm{A}}}_{\mathrm{k}} + {\hat{\mathrm{A}}}_{\mathrm{y}}\left( \mod\ 2\right)\\ {}=\left[{1}_1\left|{1}_2\right|{1}_3\left|{0}_4\right|{1}_5\left|{1}_6\right|{1}_7\left|{0}_8\right|{\mathbf{1}}_{\mathbf{9}}\left|{1}_{10}\right|\left|{\mathbf{0}}_{\mathbf{1}\mathbf{1}}\right|{\mathbf{0}}_{\mathbf{1}\mathbf{2}}\left|{\mathbf{0}}_{\mathbf{1}\mathbf{3}}\right|\dots \right]\\ {}+\left[{0}_1\left|{0}_2\right|{0}_3\left|{0}_4\right|{0}_5\left|{0}_6\right|{0}_7\left|{0}_8\right|+{\mathbf{1}}_{\mathbf{9}}\left|{0}_{10}\right|\left|{\mathbf{0}}_{\mathbf{1}\mathbf{1}}\right|{\mathbf{0}}_{\mathbf{1}\mathbf{2}}\left|{\mathbf{0}}_{\mathbf{1}\mathbf{3}}\right|\dots \right]\ \left( \mod\ 2\right)\\ {}=\left[{1}_1\left|{1}_2\right|{1}_3\left|{0}_4\right|{1}_5\left|{1}_6\right|{1}_7\left|{0}_8\right|{\mathbf{1}}_{\mathbf{9}}+{\mathbf{1}}_{\mathbf{9}}\left|{1}_{10}\right|\left|{\mathbf{0}}_{\mathbf{1}\mathbf{1}}\right|{\mathbf{0}}_{\mathbf{1}\mathbf{2}}\left|{\mathbf{0}}_{\mathbf{1}\mathbf{3}}\right|\dots \right]\ \left( \mod\ 2\right)\\ {}=\left[{1}_1\left|{1}_2\right|{1}_3\left|{0}_4\right|{1}_5\left|{1}_6\right|{1}_7\left|{0}_8\right|{\mathbf{0}}_{\mathbf{9}}\left|{1}_{10}\right|\left|{\mathbf{0}}_{\mathbf{1}\mathbf{1}}\right|{\mathbf{0}}_{\mathbf{1}\mathbf{2}}\left|{\mathbf{0}}_{\mathbf{1}\mathbf{3}}\right|\dots \right]\ \left( \mod\ 2\right)={\tilde{\mathrm{A}}}_{\mathrm{m}}.\end{array} $$

## Appendix B

Incorporation of **1**_**5**_ such as in A_j_ → Â_k_ (i.e., (6) → (11)) is achieved by A_t_, considering A_t_ = [0_1_|0_2_|0_3_|0_4_|**0**_**5**_|**0**_**6**_|**1**_**7**_|**1**_**8**_|**0**_**9**_|**1**_**10**_||**0**_**11**_|**0**_**12**_|**0**_**13**_|…] (mod 2). Here,

34 $$ \begin{array}{l}{\mathrm{A}}_{\mathrm{j}}*\ {\mathrm{A}}_{\mathrm{t}}\\ {}=\left[{1}_1\left|{1}_2\right|{1}_3\left|{0}_4\right|{\mathbf{1}}_{\mathbf{5}}\left|{\mathbf{1}}_{\mathbf{6}}\right|{\mathbf{0}}_{\mathbf{7}}\left|{\mathbf{1}}_{\mathbf{8}}\right|{\mathbf{1}}_{\mathbf{9}}\left|\left|{\mathbf{0}}_{\mathbf{1}\mathbf{0}}\right|{\mathbf{0}}_{\mathbf{1}\mathbf{1}}\right|{\mathbf{0}}_{\mathbf{1}\mathbf{2}}\Big|\dots \right]*\left[{0}_1\left|{0}_2\right|{0}_3\left|{0}_4\right|{\mathbf{0}}_{\mathbf{5}}\left|{\mathbf{0}}_{\mathbf{6}}\right|{\mathbf{1}}_{\mathbf{7}}\left|{\mathbf{1}}_{\mathbf{8}}\right|{\mathbf{0}}_{\mathbf{9}}\left|{\mathbf{1}}_{\mathbf{1}\mathbf{0}}\right|\left|{\mathbf{0}}_{\mathbf{1}\mathbf{1}}\right|{\mathbf{0}}_{\mathbf{1}\mathbf{2}}\left|{\mathbf{0}}_{\mathbf{1}\mathbf{3}}\right|\dots \right]\ \left( \mod\ 2\right)\\ {}=\left[{1}_1+{0}_1\left|{1}_2+{0}_2\right|{1}_3+{0}_3\left|{0}_4+{0}_4\right|{\mathbf{1}}_{\mathbf{5}}+{\mathbf{0}}_{\mathbf{5}}\left|{\mathbf{1}}_{\mathbf{6}}+{\mathbf{0}}_{\mathbf{6}}\right|{\mathbf{0}}_{\mathbf{7}}+{\mathbf{1}}_{\mathbf{7}}\left|{\mathbf{1}}_{\mathbf{8}}+{\mathbf{1}}_{\mathbf{8}}\right|{\mathbf{1}}_{\mathbf{9}}+{\mathbf{0}}_{\mathbf{9}}\left|{\mathbf{0}}_{\mathbf{1}\mathbf{0}}+{\mathbf{1}}_{\mathbf{1}\mathbf{0}}\right|{\mathbf{0}}_{\mathbf{1}\mathbf{1}}+{\mathbf{0}}_{\mathbf{1}\mathbf{1}}\left|{\mathbf{0}}_{\mathbf{1}\mathbf{2}}+{\mathbf{0}}_{\mathbf{1}\mathbf{2}}\right|{\mathbf{0}}_{\mathbf{1}\mathbf{3}}+{\mathbf{0}}_{\mathbf{1}\mathbf{3}}\Big|\dots \right]\ \left( \mod\ 2\right)\\ {}=\left[{1}_1\left|{1}_2\right|{1}_3\left|{0}_4\right|{\mathbf{1}}_{\mathbf{5}}\left|{1}_6\right|{1}_7\left|{0}_8\right|{1}_9\left|{1}_{10}\right|\left|{\mathbf{0}}_{\mathbf{1}\mathbf{1}}\right|{\mathbf{0}}_{\mathbf{1}\mathbf{2}}\left|{\mathbf{0}}_{\mathbf{1}\mathbf{3}}\right|\dots \right]\ \left( \mod\ 2\right) = {\hat{\mathrm{A}}}_{\mathrm{k}}\left(\mathrm{i}.\mathrm{e}.,\ (11)\right).\end{array} $$

Similarly, the change Â_k_ → Ã_m_ (i.e., (11) → (12)) is achieved by Â_y_ = [0_1_|0_2_|0_3_|0_4_|0_5_|0_6_|0_7_|0_8_|1_9_|0_10_||**0**_**11**_|**0**_**12**_|**0**_**13**_|…] (mod 2); i.e.,

35 $$ \begin{array}{l}{\hat{\mathrm{A}}}_{\mathrm{k}}*\ {\hat{\mathrm{A}}}_{\mathrm{y}}\\ {}=\left[{1}_1\left|{1}_2\right|{1}_3\left|{0}_4\right|{1}_5\left|{1}_6\right|{1}_7\left|{0}_8\right|{1}_9\left|{1}_{10}\right|\left|{\mathbf{0}}_{\mathbf{11}}\right|{\mathbf{0}}_{\mathbf{12}}\left|{\mathbf{0}}_{\mathbf{13}}\right|\dots \right]*\left[{0}_1\left|{0}_2\right|{0}_3\left|{0}_4\right|{0}_5\left|{0}_6\right|{0}_7\left|{0}_8\right|{1}_9\left|{0}_{10}\right|\left|{\mathbf{0}}_{\mathbf{11}}\right|{\mathbf{0}}_{\mathbf{12}}\left|{\mathbf{0}}_{\mathbf{13}}\right|\dots \right]\ \left( \mod\ 2\right)\\ {}=\left[{1}_1+{0}_1\left|{1}_2+{0}_2\right|{1}_3+{0}_3\left|{0}_4+{0}_4\right|{1}_5+{0}_5\left|{1}_6+{0}_6\right|{1}_7+{0}_7\left|{0}_8+{0}_8\right|{1}_9+{1}_9\left|{1}_{10}+{0}_{10}\right|\left|{\mathbf{0}}_{\mathbf{11}}+{\mathbf{0}}_{\mathbf{11}}\right|{\mathbf{0}}_{\mathbf{12}}+{\mathbf{0}}_{\mathbf{12}}\left|{\mathbf{0}}_{\mathbf{13}}+{\mathbf{0}}_{\mathbf{13}}\right|\dots \right]\ \left( \mod\ 2\right)\\ {}=\left[{1}_1\left|{1}_2\right|{1}_3\left|{0}_4\right|{1}_5\left|{1}_6\right|{1}_{\mathbf{7}}\left|{0}_{\mathbf{8}}\right|{0}_{\mathbf{9}}\left|{1}_{\mathbf{10}}\right|\left|{\mathbf{0}}_{\mathbf{11}}\right|{\mathbf{0}}_{\mathbf{12}}\left|{\mathbf{0}}_{\mathbf{13}}\right|\dots \right]\ \left( \mod\ 2\right) = {\tilde{\mathrm{A}}}_{\mathrm{m}}.\end{array} $$

The change Ã_m_ → Å_m_ (i.e., (12) → (14)) is achieved by Ã_z_, considering Ã_z_ = [0_1_|0_2_|0_3_|0_4_|0_5_|0_6_|0_7_|0_8_|1_9_|1_10_||**0**_**11**_|**0**_**12**_|**0**_**13**_|…] (mod 2). Here,

36 $$ \begin{array}{l}{\tilde{\mathrm{A}}}_{\mathrm{m}}*\ {\tilde{\mathrm{A}}}_{\mathrm{z}}\\ {}=\left[{1}_1\left|{1}_2\right|{1}_3\left|{0}_4\right|{1}_5\left|{1}_6\right|{1}_7\left|{0}_8\right|{0}_9\left|{1}_{10}\right|\left|{\mathbf{0}}_{\mathbf{11}}\right|{\mathbf{0}}_{\mathbf{12}}\left|{\mathbf{0}}_{\mathbf{13}}\right|\dots \right]*\left[{0}_1\left|{0}_2\right|{0}_3\left|{0}_4\right|{0}_5\left|{0}_6\right|{0}_7\left|{0}_8\right|{1}_9\left|{1}_{10}\right|\left|{\mathbf{0}}_{\mathbf{11}}\right|{\mathbf{0}}_{\mathbf{12}}\left|{\mathbf{0}}_{\mathbf{13}}\right|\dots \right]\ \left( \mod\ 2\right)\\ {}=\left[{1}_1+{0}_1\left|{1}_2+{0}_2\right|{1}_3+{0}_3\left|{0}_4+{0}_4\right|{1}_5+{0}_5\left|{1}_6+{0}_6\right|{1}_7+{0}_7\left|{0}_8+{0}_8\right|{0}_9+{1}_9\left|{1}_{10}+{1}_{10}\right|\left|{\mathbf{0}}_{\mathbf{11}}+{\mathbf{0}}_{\mathbf{11}}\right|{\mathbf{0}}_{\mathbf{12}}+{\mathbf{0}}_{\mathbf{12}}\left|{\mathbf{0}}_{\mathbf{13}}+{\mathbf{0}}_{\mathbf{13}}\right|\dots \right]\ \left( \mod\ 2\right)\\ {}=\left[{1}_1\left|{1}_2\right|{1}_3\left|{0}_4\right|{1}_5\left|{1}_6\right|{1}_{\mathbf{7}}\left|{0}_{\mathbf{8}}\right|{1}_{\mathbf{9}}\left|\left|{\mathbf{0}}_{\mathbf{10}}\right|{\mathbf{0}}_{\mathbf{11}}\right|{\mathbf{0}}_{\mathbf{12}}\Big|\dots \right]\ \left( \mod\ 2\right) = {\mathring{\mathrm{A}}}_{\mathrm{m}}.\end{array} $$

For an entire process, the action of A_w_ = [0_1_|0_2_|0_3_|0_4_|0_5_|0_6_|1_7_|1_8_|0_9_|0_10_||**0**_**11**_|**0**_**12**_|**0**_**13**_|…] on A_j_ gives the same result as (36):

37 $$ \begin{array}{l}{\mathrm{A}}_{\mathrm{j}}\ast {\mathrm{A}}_{\mathrm{w}}\\ {}=\left[{1}_1\left|{1}_2\right|{1}_3\left|{0}_4\right|{1}_5\left|{1}_6\right|{0}_7\left|{1}_8\right|{1}_9\left|\left|{\mathbf{0}}_{\mathbf{10}}\right|{\mathbf{0}}_{\mathbf{11}}\right|{\mathbf{0}}_{\mathbf{12}}\Big|\dots \right]*\left[{0}_1\left|{0}_2\right|{0}_3\left|{0}_4\right|{0}_5\left|{0}_6\right|{1}_7\left|{1}_8\right|{0}_9\left|\left|{\mathbf{0}}_{\mathbf{10}}\right|{\mathbf{0}}_{\mathbf{11}}\right|{\mathbf{0}}_{\mathbf{12}}\Big|\dots \right]\ \left( \mod\ 2\right)\\ {}=\left[{1}_1+{0}_1\left|{1}_2+{0}_2\right|{1}_3+{0}_3\left|{0}_4+{0}_4\right|{1}_5+{0}_5\left|{1}_6+{0}_6\right|{0}_7+{1}_7\left|{1}_8+{1}_8\right|{1}_9+{0}_9\left|\left|{\mathbf{0}}_{\mathbf{10}}+{\mathbf{0}}_{\mathbf{10}}\right|{\mathbf{0}}_{\mathbf{11}}+{\mathbf{0}}_{\mathbf{11}}\right|{\mathbf{0}}_{\mathbf{12}}+{\mathbf{0}}_{\mathbf{12}}\Big|\dots \right]\ \left( \mod\ 2\right)\\ {}=\left[{1}_1\left|{1}_2\right|{1}_3\left|{0}_4\right|{1}_5\left|{1}_6\right|{1}_{\mathbf{7}}\left|{0}_{\mathbf{8}}\right|{1}_{\mathbf{9}}\left|\left|{\mathbf{0}}_{\mathbf{10}}\right|{\mathbf{0}}_{\mathbf{11}}\right|{\mathbf{0}}_{\mathbf{12}}\Big|\dots \right]\ \left( \mod\ 2\right) = {\mathring{\mathrm{A}}}_{\mathrm{m}}.\ \end{array} $$

## Appendix C

For A_d_ such that A_d_ = [1_1_|0_2_|1_3_|1_4_|…] (mod 2),

so that the index numbers are the same, 0 s are incorporated:

$$ \begin{array}{l}{\mathrm{A}}_{\mathrm{c}}\to {\mathrm{A}}_{\mathrm{c}1}=\left[{1}_1\left|{1}_2\right|{0}_3\left|{0}_4\right|{1}_5\left|{1}_6\right|{0}_7\left|{1}_8\right|\left|{\mathbf{0}}_{\mathbf{9}}\right|\left|{\mathbf{0}}_{\mathbf{1}\mathbf{0}}\right|{\mathbf{0}}_{\mathbf{1}\mathbf{1}}\left|{\mathbf{0}}_{\mathbf{1}\mathbf{2}}\right|\dots \right]\ \left( \mod\ 2\right),\\ {}{\mathrm{A}}_{\mathrm{d}}\to {\mathrm{A}}_{\mathrm{d}1}=\left[{\mathbf{0}}_{\mathbf{1}}\left|{\mathbf{0}}_{\mathbf{2}}\right|{\mathbf{0}}_{\mathbf{3}}\left|{\mathbf{0}}_{\mathbf{4}}\right|{\mathbf{0}}_{\mathbf{5}}\left|{\mathbf{0}}_{\mathbf{6}}\right|{\mathbf{0}}_{\mathbf{7}}\left|{\mathbf{0}}_{\mathbf{8}}\right|\left|{1}_{1+8}\right|{0}_{2+8}\left|{1}_{3+8}\right|{1}_{4+8}\left|{\mathbf{0}}_{\mathbf{1}\mathbf{3}}\right|{\mathbf{0}}_{\mathbf{1}\mathbf{4}}\left|{\mathbf{0}}_{\mathbf{1}\mathbf{5}}\right|\dots \right]\ \left( \mod\ 2\right).\\ {}{\mathrm{A}}_{\mathrm{e}}=\left\{{\mathrm{A}}_{\mathrm{c}},\ {\mathrm{A}}_{\mathrm{d}}\right\}\ \left( \mod\ 2\right) = \left[{1}_1\left|{1}_2\right|{0}_3\left|{0}_4\right|{1}_5\left|{1}_6\right|{0}_7\left|{1}_8\right|{1}_9\left|{0}_{10}\right|{1}_{11}\Big|{1}_{12}\left|{\mathbf{0}}_{\mathbf{1}\mathbf{3}}\right|{\mathbf{0}}_{\mathbf{1}\mathbf{4}}\left|{\mathbf{0}}_{\mathbf{1}\mathbf{5}}\right|\dots \right]\ \left( \mod\ 2\right)\\ {}=\left[{\mathrm{A}}_{\mathrm{c}}\left|{\mathrm{A}}_{\mathrm{d}}\right|\dots \right]\ \left( \mod\ 2\right).\end{array} $$

## Appendix D

$$ \begin{array}{l}\mbox{\fontencoding{T1}\fontfamily{cmr}\selectfont\char"0E} {\hat{\mathrm{A}}}_{\mathrm{k}}\mbox{\fontencoding{T1}\fontfamily{cmr}\selectfont\char"0F} *\mbox{\fontencoding{T1}\fontfamily{cmr}\selectfont\char"0E} {\hat{\mathrm{A}}}_{\mathrm{y}}\mbox{\fontencoding{T1}\fontfamily{cmr}\selectfont\char"0F} =\mbox{\fontencoding{T1}\fontfamily{cmr}\selectfont\char"0E} {\hat{\mathrm{A}}}_{\mathrm{k}}\mbox{\fontencoding{T1}\fontfamily{cmr}\selectfont\char"0F} +\mbox{\fontencoding{T1}\fontfamily{cmr}\selectfont\char"0E} {\hat{\mathrm{A}}}_{\mathrm{y}}\mbox{\fontencoding{T1}\fontfamily{cmr}\selectfont\char"0F} \\ {}=\left[{2}_1\left|{5}_2\right|{3}_3\left|{0}_4\right|{3}_5\left|{6}_6\right|{2}_7\left|{0}_8\right|{\mathbf{4}}_{\mathbf{9}}\left|{1}_{10}\right|\left|{\mathbf{0}}_{\mathbf{11}}\right|{\mathbf{0}}_{\mathbf{12}}\left|{\mathbf{0}}_{\mathbf{13}}\right|\dots \right]\\ {}+\left[{0}_1\left|{0}_2\right|{0}_3\left|{0}_4\right|{0}_5\left|{0}_6\right|{0}_7\left|{0}_8\right|{\mathbf{3}}_{\mathbf{9}}\left|{0}_{10}\right|\left|{\mathbf{0}}_{\mathbf{11}}\right|{\mathbf{0}}_{\mathbf{12}}\left|{\mathbf{0}}_{\mathbf{13}}\right|\dots \right]\ \left( \mod\ 7\right)\\ {}=\left[{2}_1\left|{5}_2\right|{3}_3\left|{0}_4\right|{3}_5\left|{6}_6\right|{2}_7\left|{0}_8\right|{\mathbf{4}}_{\mathbf{9}}+{\mathbf{3}}_{\mathbf{9}}\left|{0}_{10}\right|\left|{\mathbf{0}}_{\mathbf{11}}\right|{\mathbf{0}}_{\mathbf{12}}\left|{\mathbf{0}}_{\mathbf{13}}\right|\dots \right]\ \left( \mod\ 7\right)\\ {}=\left[{2}_1\left|{5}_2\right|{3}_3\left|{0}_4\right|{3}_5\left|{6}_6\right|{2}_7\left|{0}_8\right|{\mathbf{0}}_{\mathbf{9}}\left|{1}_{10}\right|\left|{\mathbf{0}}_{\mathbf{11}}\right|{\mathbf{0}}_{\mathbf{12}}\left|{\mathbf{0}}_{\mathbf{13}}\right|\dots \right]\ \left( \mod\ 7\right)\\ {} = \mbox{\fontencoding{T1}\fontfamily{cmr}\selectfont\char"0E} {\tilde{\mathrm{A}}}_{\mathrm{m}}\mbox{\fontencoding{T1}\fontfamily{cmr}\selectfont\char"0F} .\end{array} $$

## Appendix E

$$ \begin{array}{l}\mbox{\fontencoding{T1}\fontfamily{cmr}\selectfont\char"0E} {\tilde{\mathrm{A}}}_{\mathrm{m}}\mbox{\fontencoding{T1}\fontfamily{cmr}\selectfont\char"0F} *\mbox{\fontencoding{T1}\fontfamily{cmr}\selectfont\char"0E} {\tilde{\mathrm{A}}}_{\mathrm{z}}\mbox{\fontencoding{T1}\fontfamily{cmr}\selectfont\char"0F} \\ {}=\left[{2}_1\left|{5}_2\right|{3}_3\left|{0}_4\right|{3}_5\left|{6}_6\right|{2}_7\left|{0}_8\right|{\mathbf{0}}_{\mathbf{9}}\left|{1}_{10}\right|\left|{\mathbf{0}}_{\mathbf{11}}\right|{\mathbf{0}}_{\mathbf{12}}\left|{\mathbf{0}}_{\mathbf{13}}\right|\dots \right]+\left[{0}_1\left|{0}_2\right|{0}_3\left|{0}_4\right|{0}_5\left|{0}_6\right|{0}_7\left|{0}_8\right|{1}_9\Big|\left|{6}_{10}\right|\left|{\mathbf{0}}_{\mathbf{11}}\right|{\mathbf{0}}_{\mathbf{12}}\left|{\mathbf{0}}_{\mathbf{13}}\right|\dots \right]\\ {}\left( \mod\ 7\right)\\ {}=\left[{2}_1+{0}_1\left|{5}_2+{0}_2\right|{3}_3+{0}_3\left|{0}_4+{0}_4\right|{3}_5+{0}_5\left|{6}_6+{0}_6\right|{2}_7+{0}_7\left|{0}_8+{0}_8\right|{0}_9+{1}_9\left|{1}_{10}+{6}_{10}\right|{\mathbf{0}}_{\mathbf{11}}+{\mathbf{0}}_{\mathbf{11}}\right|{\mathbf{0}}_{\mathbf{12}}+{\mathbf{0}}_{\mathbf{12}}\\ {}\left|{\mathbf{0}}_{\mathbf{13}}+{\mathbf{0}}_{\mathbf{13}}\right|\dots \Big]\ \left( \mod\ 7\right)\\ {} = \left[{2}_1\left|{5}_2\right|{3}_3\left|{0}_4\right|{3}_5\left|{6}_6\right|{2}_7\left|{0}_8\right|{1}_9\left|\left|{\mathbf{0}}_{\mathbf{10}}\right|{\mathbf{0}}_{\mathbf{11}}\right|{\mathbf{0}}_{\mathbf{12}}\Big|\dots \right]\ \left( \mod\ 7\right)=\mbox{\fontencoding{T1}\fontfamily{cmr}\selectfont\char"0E} {\mathring{A}}_{\mathrm{m}}\mbox{\fontencoding{T1}\fontfamily{cmr}\selectfont\char"0F} .\end{array} $$

## Appendix F

$$ \begin{array}{l}\mbox{\fontencoding{T1}\fontfamily{cmr}\selectfont\char"0E} {\mathrm{A}}_{\mathrm{j}}\mbox{\fontencoding{T1}\fontfamily{cmr}\selectfont\char"0F} *\mbox{\fontencoding{T1}\fontfamily{cmr}\selectfont\char"0E} {\mathrm{A}}_{\mathrm{w}}\mbox{\fontencoding{T1}\fontfamily{cmr}\selectfont\char"0F} \\ {}=\left[{2}_1\left|{5}_2\right|{3}_3\left|{0}_4\right|{6}_5\left|{2}_6\right|{0}_7\left|{4}_8\right|{1}_9\left|\left|{\mathbf{0}}_{\mathbf{10}}\right|{\mathbf{0}}_{\mathbf{11}}\right|{\mathbf{0}}_{\mathbf{12}}\Big|\dots \right] + \left[{0}_1\left|{0}_2\right|{0}_3\left|{0}_4\right|{4}_5\left|{4}_6\right|{2}_7\left|{3}_8\right|{0}_9\left|\left|{\mathbf{0}}_{\mathbf{10}}\right|{\mathbf{0}}_{\mathbf{11}}\right|{\mathbf{0}}_{\mathbf{12}}\Big|\dots \right]\ \Big( \mod \\ {}7\Big) = \left[{2}_1\left|{5}_2\right|{3}_3\left|{0}_4\right|{3}_5\left|{6}_6\right|{2}_7\left|{0}_8\right|{1}_9\left|\left|{\mathbf{0}}_{\mathbf{10}}\right|{\mathbf{0}}_{\mathbf{11}}\right|{\mathbf{0}}_{\mathbf{12}}\Big|\dots \right]\ \left( \mod\ 7\right)=\mbox{\fontencoding{T1}\fontfamily{cmr}\selectfont\char"0E} {\mathring{\mathrm{A}}}_{\mathrm{m}}\mbox{\fontencoding{T1}\fontfamily{cmr}\selectfont\char"0F} .\end{array} $$
